# Genetic and Morphological Diversity in Spontaneous Populations of *Brassica rapa*: How Do Feral Populations Differ From Wild Ones?

**DOI:** 10.1111/mec.70461

**Published:** 2026-07-08

**Authors:** L. Gay, S. Boitard, L. Bousset‐Vaslin, A.‐M. Chèvre, G. Deniot, C. Falentin, M. Gautier, S. Geneste, M. Tiret, J. Ronfort

**Affiliations:** ^1^ UMR AGAP Institut Université de Montpellier, CIRAD, INRAE, Institut Agro Montpellier France; ^2^ UMR CBGP, INRAE, CIRAD, IRD, Montpellier SupAgro Univ Montpellier Montpellier France; ^3^ UMR IGEPP, INRAE, Institut Agro Université de Rennes Le Rheu France

**Keywords:** *Brassica rapa*, crop wild relatives, feralisation, population genomics

## Abstract

Crop wild relatives are a valuable source of genetic material for improving cultivated crops. However, feral populations—self‐sustaining plants that have escaped cultivation—are often mistaken for wild relatives because they exhibit traits such as seed dispersal and dormancy, which resemble wild forms and support persistence in natural habitats. This study examined the genetic diversity, population structure, and morphology of 117 *Brassica rapa* populations, including landraces from farms or genebanks and spontaneous populations sampled in the wild across the Mediterranean basin. Substantial genetic diversity was observed in the 45 wild populations from Italy and Algeria, with clear genetic differentiation from cultivated landraces. In contrast, spontaneous populations in France and Slovenia showed reduced diversity, comparable to that of landraces, and clustered closely with them in genetic analyses. Population trees and admixture analyses showed that the French spontaneous populations were more closely related to other landraces than to any other wild populations, which confirmed their feral origin. We also identified the most likely landrace ancestor for each feral population, which pointed towards a French landrace in nearly all cases. These findings support previous evidence that most European *B. rapa* populations are feral rather than truly wild. Morphological analyses further showed that feral populations displayed traits intermediate between wild and cultivated groups and even suggested some reversion towards pre‐domestication traits in root morphology and germination. This highlights how feralisation can blur distinctions between wild relatives and crop escapes. Overall, the study emphasizes the need to disentangle wild and feral populations when identifying genetic resources for breeding. Further research is required to clarify the history of feralisation in *B. rapa* , including the origins and spread of populations, gene flow with crops and wild relatives, and the role of local adaptation in shaping their persistence.

## 1 Introduction

Crop wild relatives (CWRs) are wild species or taxa related to a crop, whether wild forms of the same species, crop progenitors or other species closely related to the cultivated taxa (Maxted et al. 2006). As these species have not undergone the genetic bottleneck of domestication, they are a valuable source of genetic diversity for improving their cultivated relatives (Maxted and Kell 2009), especially to adapt crops to changing environments (Esquinas‐Alcázar 2005; Hajjar and Hodgkin 2007; Guarino and Lobell 2011; Nair 2019). CWRs have therefore been used for over 100 years in crop improvement programmes, particularly to increase resistance to biotic and abiotic stresses (Cockel et al. 2022). However, many of these species could be severely impacted by climate change, with a reduction or fragmentation of their distribution range (Jarvis et al. 2008; Brehm et al. 2016; Aguirre‐Gutiérrez et al. 2017). A detailed knowledge of their distribution and diversity is therefore crucial.

A common caveat in the study of crop wild relatives is the confusion with feral populations. Feral plant populations occur when crop plants escape cultivation and establish self‐sustaining and stable populations in natural environments (Gressel 2005; Bagavathiannan and Van Acker 2008; Scossa and Fernie 2021). Yet, while some feral populations can persist for years, many seem to be transient. For example, in a survey of *Brassica* populations in New Zealand, about 60% of populations disappeared within 2 years (Meffin et al. 2015). The confusion with wild relatives stems from the fact that feral plants generally differ from their cultivated counterparts by a set of traits that resemble the wild forms and allow them to persist in the wild, such as seed dispersal and dormancy (Gressel 2005; Bagavathiannan and Van Acker 2008; Wu et al. 2021). The degree to which feral plants resemble wild forms varies according to the extent of “de‐domestication” after escape from cultivation (Gressel 2005; Gering et al. 2019; Mabry et al. 2023).

In terms of genetic diversity, however, feral populations are expected to resemble the crop species from which they originated. On average, they have reduced genetic diversity compared to their crop wild relatives, which have not undergone the genetic bottleneck of domestication (Maxted and Kell 2009). Nevertheless, feral populations can diverge from their domesticated ancestors over time and may in particular adapt to local conditions (Qi et al. 2017; Mittell et al. 2020). The amount of genetic differentiation from their crop species of origin is thus expected to vary according to both the age of the feral population and the level of gene flow with the crop. Feral populations of crops such as oilseed rape, for example, show significant genetic differentiation from commercial varieties, with less than 50% shared alleles (Pascher et al. 2010). These differentiations suggest that feral populations could represent a genetic resource alongside crop wild relatives (Mabry et al. 2023).

Multiple pathways lead to the evolution of feral plant populations. Feral populations often arise from seed immigration from nearby fields or through persistent seed banks. This accounts for 35%–40% of feral oilseed rape populations, for example (Pivard et al. 2008). Such feral populations entirely derive from a domesticated progenitor, with almost no admixture, and are called endoferals (Gressel 2005). Alternatively, exoferalisation results from the crossbreeding between wild and cultivated forms (Bagavathiannan and Van Acker 2008). Both mechanisms are common and occur with similar frequency in plants (Gering et al. 2019).

Feral populations of rice are perhaps the best‐studied examples of feralisation (Ishikawa et al. 2005; Cao et al. 2006; Londo and Schaal 2007; He et al. 2017; Li et al. 2017; Qiu et al. 2017, 2020; Sun et al. 2019). However, it has recently been shown that feralisation may be more common than previously thought, with evidence reported for over 45 crop species (Mabry et al. 2023). As well as providing an opportunity to study evolution in action, the study of feral populations could help us to better understand how their establishment can alter local ecosystems, potentially affecting native species and agricultural practices (Garnier et al. 2006). Indeed, a substantial proportion of feral populations have become weeds or invasive species (Ellstrand et al. 2010). Feral populations could also pose problems for containing novel traits, particularly in the context of genetically modified crops, as they could act as a bridge between crops and wild plants (Bagavathiannan and Van Acker 2008).

*Brassica rapa* L. (*Brassicaceae*, 2*n* = 20) is a worldwide‐grown allogamous species comprising various types of turnips and leafy greens (bok choy, rapini, grelos, etc.) as well as oilseed crops (turnip rape, toria, yellow sarsons) and weedy forms ( *B. rapa ssp. sylvestris* ; McAlvay et al. 2021). These morphotypes of *B. rapa* are identified as different sub‐species and have been selected for different characters (enlarged tap root, rosette morphology, different leaf shapes and structures, seed size and oil content; Zhao et al. 2010). The species is largely described as self‐incompatible with the exception of yellow sarsons, and many sub‐species are annual, though turnip rape cultivars and some rapa cabbage are biennial and require vernalization to induce flowering (Zhao et al. 2010). The morphological diversity of this species makes it a relevant model to study domestication and artificial selection (Bird et al. 2017; Qi et al. 2017), which is now facilitated by the availability of a well‐annotated genome (Wang et al. 2011; Belser et al. 2018) and pan‐genome (Cai et al. 2022). *B. rapa* (2*n* = 20) is the donor of the A genome (haploid size around 529 Mb, Johnston et al. 2005) of oilseed rape ( *B. napus* , AACC genome, 2*n* = 4*X* = 38) which resulted from the crossing with *B. oleracea* (cabbage, C genome, 2*n* = 18). It is also a nutritionally important crop (Zhang et al. 2024), providing a valuable source of vitamin C, protein, secondary metabolites and minerals for humans and animals (De Pascale et al. 2007; Zhao et al. 2010; Zou et al. 2021) and the genus *Brassica* as a whole was valued at around 90 million dollars in 2023 (FAOSTAT 2023).

The domestication of the diverse *Brassica rapa* crops has long been thought to have occurred in the Mediterranean basin around 3500 bc (Candolle 2011), and the turnip morphotype is believed to be the first one domesticated. More recently, several studies used molecular data to reconstruct the domestication and diversification history of this crop (Crouch et al. 1995; Andersen et al. 2009; Zhao et al. 2010; Cheng et al. 2016; Tanhuanpää et al. 2016). However, including accessions that were sampled in the wild but are likely to be feral has led to misleading conclusions about domestication (Guo et al. 2014), particularly concerning the centre of domestication. Indeed, more recent studies have shown that turnips and oilseeds were first domesticated in the mountains of Central Asia between 3430 and 5930 years ago. They were then further selected as leafy vegetables and oil crops in the Mediterranean region and East Asia (McAlvay et al. 2021). Genetic data and written records also show that the eastward introduction and diversification of *B. rapa* happened 2400–4100 years ago (Qi et al. 2017).

Feralisation is frequent in the *Brassica* genus. For example, feral populations of oilseed rape are widespread and have been particularly studied, mainly because of their potential role in gene flow from genetically modified crops (Ellstrand et al. 1999; Bagavathiannan and Van Acker 2008). Feral populations also occur in *Brassica oleracea* (Maggioni et al. 2020; Mabry, Turner‐Hissong, et al. 2021). In *Brassica rapa* , Crouch et al. (1995) used RFLP markers and first suggested that wild accessions sampled in the wild in America (both North and South, California and Argentina) had been introduced recently, likely from feral populations. In northern Europe, the populations sampled in the wild were described as true wild and not ferals, because they showed a clear differentiation from cultivated subspecies on ISSR markers (Andersen et al. 2009). Yet, turnip morphotypes were not included in the analyses and the “wild” populations had low levels of diversity, similar to the cultivated populations. Their status was therefore unclear. Later studies confirmed that non‐crop samples occurring in the Americas and much of Europe are feral (McAlvay et al. 2021; Saban et al. 2023).

These pieces of evidence for feral populations were mostly based on comparisons with a limited number of truly wild accessions. Actually, few truly wild populations of *Brassica rapa* have been described in the literature, and identifying truly wild relatives of *B. rapa* can be hindered by the widespread presence of feral populations. Several authors, however, agree that true wild populations can be found in Italy (Crouch et al. 1995; McAlvay et al. 2021), Algeria (Crouch et al. 1995; Guo et al. 2014; Aissiou et al. 2018), as well as in the Caucasus and Siberia (McAlvay et al. 2021). Only one study examined the genetic diversity of wild *B. rapa* at the population level in Algeria (Aissiou et al. 2018). There, *B. rapa* wild populations grow in fields and roadsides and are considered weeds. They display high levels of diversity at SSR markers and form a cluster distinct from Algerian landraces, regardless of their geographical origin.

Mistaking feral populations for wild forms because they resemble them morphologically and grow in non‐managed areas can lead to an underestimation of the genetic diversity of wild relatives. This occurred in *B. rapa* , where the genetic diversity of wild forms was underestimated (Crouch et al. 1995). Therefore, distinguishing feral from truly wild populations and accurately describing the diversity of the latter is crucial in order to direct conservation efforts towards them, especially in the context of climate change.

In the present study, we analyse a large collection of 117 *B. rapa* populations, which were sampled in cultivated fields (landraces) or in the wild (hereafter referred to as “spontaneous”, comprising potentially wild or feral populations) in five countries of the Mediterranean basin (France, Italy, Slovenia, Algeria, Tunisia). We aim at describing the genetic diversity of these populations and the relationships among them. Ultimately, we address the subject of feralisation and attempt to give insights into the history of putatively feral populations in our sample.

## 2 Material and Methods

### 2.1 Population Sampling

62 “spontaneous” populations of *B. rapa* were collected in the wild in Algeria (28), Italy (17), France (16), and Slovenia (1). The type of land use at the sites where the populations were sampled was recorded. This included cultivated fields (of annual or perennial crops), pastures, wastelands or fallows, field borders, and semi‐natural areas such as those found alongside rivers, ponds, or in public gardens (Table S1). Siliques were collected from up to 30 plants per population, depending on population size and accessibility. 55 *B. rapa* landraces were collected either through direct collects in farms for Algeria (22), Tunisia (5), and Italy (5) or through Biological Resource Centers (BRC) maintaining old landraces in France (BRC BrACySol, 21) and Slovenia (BRC KIS, 2). Most collected landraces were turnips (ssp. *rapa*), except for a few broccoletto (ssp. *sylvestris* var. *esculenta*) selected by Italian farmers. Protocols for population sampling and controls to eliminate potential errors in species identification (including flow cytometry, chromosome counting, and sequencing of a chloroplast region) are detailed in Falentin et al. (2024).

To multiply the seeds and avoid maternal effects, we produced seeds from 10 plants per population under pollen‐proof cages (two cages with five plants each, Tiret et al. 2025) at a single location (Le Rheu, France, 48°06′N 1°47′O).

### 2.2 Whole Genome Resequencing

Tissue samples from 30 plants per population, representing 11–30 mother plants collected in situ, were pooled for DNA extraction. Libraries from the DNA pools were constructed following the Illumina procedures and were sequenced with Illumina HiSeq technology (see details in Tiret et al. 2025). We targeted ~50× pool coverage.

Raw paired‐end reads were cleaned as detailed in Tiret et al. (2025) and with fastp v.0.20.1 (Chen et al. 2018) using default options to remove contaminant adapter sequences and trim for poor quality bases (i.e., with a Phred‐quality score < 15). Cleaned reads were mapped to the *B. rapa C1.3 var rapifera* reference genome (Maillet et al. 2025; Tiret et al. 2025) using bwa‐ mem 0.7.17 (Li and Durbin 2009; Li 2013). Reads with a mapping quality Phred‐score < 20 or duplicates were removed using the view (option *‐q 20*) and markdup programs from the SAMtools v. 1.14 (Danecek et al. 2021). Variants were called with Freebayes v1.3.1 (Garrison and Marth 2012) with the options *‐K* (*aka* pooled‐continuous) *‐C 1 ‐F 0.01* as recommended when analysing PoolSeq data. In addition, we used the options *‐m 30* and *‐q 20* to set the minimum mapping quality for an alignment to be used to 30 and the minimum base quality for a base to be considered to 20. Finally, clumping was disabled (option *‐E −1*) to have as many simple variants as possible and we retained only biallelic SNPs supported by at least five reads for the minor allele across samples (*‐G 5*). To improve computational efficiency, variant calling was performed in parallel on 7550 non‐overlapping 50‐kb windows. Resulting vcf files were concatenated and reordered using the concat and view programs of bcftools (v1.20, Danecek et al. 2021), filtering low quality variant with the option *‐i QUAL > 20*. Finally, the vcf was parsed and further filtered using the vcf2pooldata function of the R package poolfstat (Gautier et al. 2022), removing SNPs within 5 bp of indels and setting a minimum allele frequency (MAF) of 10^−3^. The final dataset comprised 26,139,409 SNPs for 117 pools across the 10 chromosomes of *B. rapa* and 670 unanchored scaffolds.

### 2.3 Levels of Diversity and Population Structure

Nucleotide diversity (π) was estimated with *npstat v.1* from *bam* alignment files (Ferretti et al. 2013) using 10‐kb sliding windows. The results were filtered to only retain windows that were covered by at least one read over 9000 positions. Mean π across windows was calculated for each group of populations, sampled in the wild or landraces for each country and differences were tested using Tukey tests.

Private alleles were examined by creating subgroups of populations using poolfstat utilities. Based on the diversity results, we divided the spontaneous populations into two subgroups: ‘wild’ (wild populations from Italy and Algeria, hereafter referred to as WI and WA, respectively) and ‘feral’ (spontaneous populations with a similar level of diversity to landraces, but sampled in the wild in France and Slovenia, hereafter WF and WS). The landraces formed a third subgroup labelled ‘landraces’, comprising populations from Algeria, France, Italy, Slovenia and Tunisia (hereafter LA, LF, LI, LS and LT). For each subgroup, variants with extreme overall coverage (outside the 1st and 99th percentiles) and those with overall MAF < 1% were excluded. The set of private alleles was compared between subgroups using an upset plot with the R package UpSetR (v1.4.0, Conway et al. 2017), which allowed identifying private alleles for each subgroup and the intersection of subgroups.

Genetic structure was explored using a ‘random allele’ Principal Component Analysis (PCA) (randomallele.pca() function in the poolfstat package) and pairwise $F_{\mathrm{ST}}$ estimates, using the compute.fstats() function from the poolfstat package in R. Additional analyses (including DAPC and count of the number of polymorphic sites) were used to build nested core‐collections maximizing diversity (detailed in Text S1).

### 2.4 Origin of Spontaneous Populations: *F*‐Statistics and Admixture

To investigate admixture, we estimated the *F*‐statistics (Patterson et al. 2012) for all triplets and quadruplets of populations among the 117 sampled. We used the *compute.fstats* function in the R package *poolfstat* (Gautier et al. 2022) with the default options, defining blocks of 200,000 consecutive SNPs for the block‐jackknife estimation of standard errors (SE) and *Z*‐scores.

We first applied *F*_3_‐based treeness tests to identify admixed populations. Admixed populations showing significantly negative *F*_3_ statistics at the 95% threshold: *Z*‐score < −1.65 were excluded from subsequent analyses.

Secondly, we used *F*_4_ statistics to conduct tests of treeness among quadruplets of populations, to clarify relationships between landraces and spontaneous populations (Text S2). Initially, we tested whether the Italian and Algerian spontaneous populations (WI and WA) were closer to each other than to any landrace (including those from the same country, LI and LA). To do this, we focused on all possible population quadruplets consisting of one WI, one WA, and two landraces (Lx and Ly), and considered all the calculated $F_{4}$ statistics for the three configurations that represent all possible unrooted topologies: (WI, WA; Lx, Ly), (WI, Lx; WA, Ly), and (WI, Ly; WA, Lx). Note that permutations within each pair are equivalent and yield $F_{4}$ values identical in absolute value (e.g., 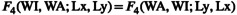 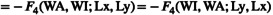). Under the hypothesis of a closer proximity between WI and WA than with any other landrace, we expect the configuration (WI, WA; Lx, Ly) to have the lowest $F_{4}$ (in absolute value) and even pass the treeness test (i.e., $F_{4}\left(\mathrm{WI},\mathrm{WA};\mathrm{Lx},\mathrm{Ly}\right)=0$ and $\left|Z\right|<1.96$ at the 95% confidence threshold) in the absence of significant gene flow between the compared landrace and spontaneous populations since their split. Conversely, the two alternative configurations should never pass the treeness test (i.e., $F_{4}\left(\mathrm{WI},\mathrm{Lx};\mathrm{WA},\mathrm{Ly}\right)\neq 0$ and $F_{4}\left(\mathrm{WI},\mathrm{Ly};\mathrm{WA},\mathrm{Lx}\right)\neq 0$). We then applied the same reasoning to the French spontaneous populations (WF): if they were truly wild, we would expect the French spontaneous populations to be closest to the Algerian or Italian wild populations, and more distant from the landraces, due to the domestication bottleneck. To formally test this, we then focussed on all possible quadruplets including one spontaneous population from France (WF), one from Algeria (WA) and one from Italy (WI), as well as one landrace (Lx). We compared the Z‐scores for the three configurations of a given quadruplet to verify whether the configuration (WF, Lx; WA, WI) gave the lowest $\left|F_{4}\right|$ estimates and tended to pass the treeness test more often than alternative configurations. However, it must be noted that if this treeness test confirms that the French spontaneous populations are closer to the landraces than to the Algerian or Italian wild populations, there are two possible scenarios: either the French spontaneous populations are feral, or they are the closest wild ancestors of the landraces. To distinguish between these scenarios, we tested the hypothesis that the French spontaneous populations are derived from the French landraces. We created all possible quadruplets involving a WF population, a wild population (WA or WI, hereafter referred to as Wx), a French landrace (LF) and a landrace from another country (Ly). If WF was a proxy for the wild ancestor of the landraces, then the configuration (WF, Wx; LF, Ly) should pass the treeness test (i.e., $F_{4}\left(\mathrm{WF},\mathrm{Wx};\mathrm{LF},\mathrm{Ly}\right)=0$). Conversely, if WF was feral and more closely related to LF than to any other landrace from a more distant region (Ly), then $F_{4}\left(\mathrm{WF},\mathrm{LF};\mathrm{Wx},\mathrm{Ly}\right)$ should be null. Slovenian spontaneous populations were analysed similarly.

Finally, the closest landrace proxy for each feral population (17 French and Slovenian spontaneous populations) was identified using the $F_{3}$ statistics. Indeed, $F_{3}\left(\mathrm{Wx};{\mathrm{WF}}_{t},\mathrm{Ly}\right)$ measures the genetic distance between a wild population Wx and the ancestor of the group (${\mathrm{WF}}_{t}$, Ly) formed by the target feral population ${\mathrm{WF}}_{t}$ and a landrace (Ly). For a given ${\mathrm{WF}}_{t}$ and Wx, the closest landrace proxy may thus be considered as the one maximizing $F_{3}\left(\mathrm{Wx};{\mathrm{WF}}_{t},\mathrm{Ly}\right)$ (see Text 2 for a visual interpretation of the $F_{3}$). For each feral population, we determined which landraces maximize the $F_{3}$ for each of the wild ancestors (out of the 44 WI and WA) and selected as proxy the one that was detected the most frequently.

### 2.5 Morphological Measurements in Common Garden

A common garden experiment was conducted at Le Rheu (France, 48°06′N 1°47′O). The 117 populations were sown in mid‐September 2022 in an alpha lattice design with three repetitions, using 150 seeds per plot of 1 m × 1.5 m.

Five plants per plot were measured for each trait, yielding 15 measurements per population. Traits included root and fourth‐leaf dry weight, specific leaf area (SLA) of the fourth leaf, and leaf number. Following the International Board for Plant Genetic Resources criteria (IBPGR and CEC 1990), roots were scored for shape (criteria 4.2.82), external (4.2.92) and internal colour (4.2.93), and number of lateral roots (4.2.98). Additionally, each population received a mark describing its basitony, that is, the level of “bushiness” of the above‐ground organs. After maturity, up to five plants per plot were harvested to collect the pods and thresh them in bulks. We weighted the total mass of seeds produced in each bulk and measured the thousand seeds weight. The total number of seeds per plot was estimated as the total mass divided by the average seed weight, deduced from the thousand seeds weight. Finally, the average number of seeds produced per plant was calculated as the total number of seeds divided by the number of plants harvested.

To visualize how the different populations differed morphologically, we used a Multiple Correspondence analysis (MCA) on the categorical variables (colour and shape of the root) and a PCA on quantitative variables, including the coordinates on the first three axes of the MCA, using the R package FactoMineR (Le et al. 2008). Finally, we tested for significant differences between landraces and populations sampled in the wild. For quantitative traits (root dry weight, leaf dry weight, leaf number, SLA, seed number and thousand seeds weight), we fitted a linear mixed model using the R package lme4 (v1.1‐30, Bates et al. 2015; Equation (1)) with population and replicate as random effects, and country as a fixed effect. We also included a fixed effect ‘type’ aimed at contrasting landraces, ‘true’ wild populations from Italy and Algeria and spontaneous populations from France and Slovenia suspected to be of feral origin based on genetic results.

(1) $${\text{Trait}}_{\text{ijklm}}=\mu +{\text{type}}_{j}+{\text{country}}_{k}+{\mathrm{pop}}_{l}+{\text{replicate}}_{m}+e_{\text{ijklm}},$$

We tested for the significance of ‘type’ using a likelihood ratio test, followed by pairwise comparisons of estimated marginal means. For qualitative traits (basitony, lateral roots, colour and shape of the root), we used pairwise *χ* ^2^ tests, with medians across the 15 plants measured per population to avoid pseudo‐replication. Finally, we tested for an effect of the type of land use at the sites where the populations were sampled (detailed in Table 1) on root characteristics using the same tests as described above (linear mixed model for root dry weight and *χ* ^2^ tests for lateral roots).

## 3 Results

### 3.1 Levels of Genetic Diversity

**FIGURE 1:**
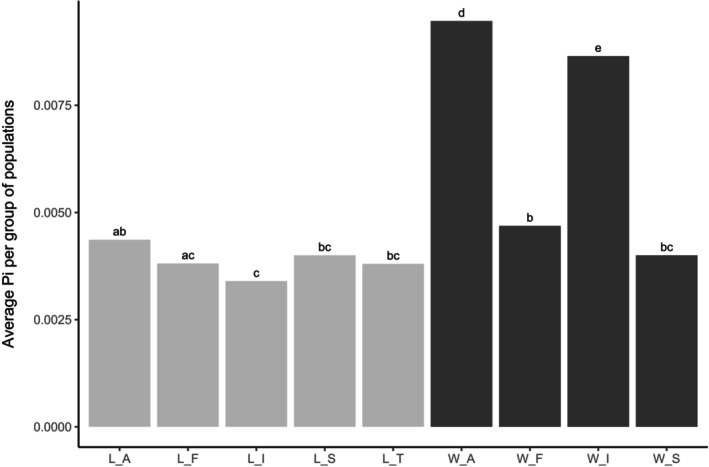
Boxplot of π, the average number of pairwise differences among sequences for landraces (“L_” in light grey) and spontaneous populations sampled in the wild (“W_” in dark grey). The *x*‐axis shows the countries of origin of the populations, A for Algeria, F for France, I for Italy, S for Slovenia and T for Tunisia. The letters indicate the groups that are significantly different based on Tukey tests.

Spontaneous populations sampled in the wild in Algeria and Italy both exhibit higher levels of genetic diversity than all other populations, including all landraces (Figure 1). This suggests that they have not experienced any domestication bottlenecks. In contrast, spontaneous populations sampled in the wild in France and Slovenia exhibit lower levels of diversity that are similar to those observed in their domesticated counterparts.

**FIGURE 2:**
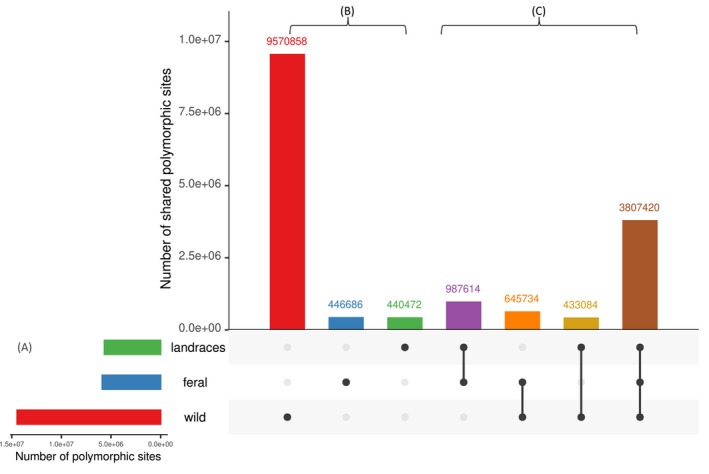
Upset plot representing the number of SNP among the SNPs ascertained across the 117 populations that are polymorphic in three population subsets: Landraces from Algeria, France, Italy, Slovenia and Tunisia; spontaneous populations sampled in the wild in France and Slovenia, labelled as ‘feral’ due to their low diversity in Figure 1; spontaneous populations from Italy and Algeria, labelled as ‘wild’. (A) Total number of alleles, (B) number of private alleles and (C) number of alleles shared among groups.

The number of polymorphic sites with coverage between the 1st and 99th percentiles and an overall MAF > 1% was more than 2.5 times higher in the ‘wild’ group (WA and WI) than in the ‘landrace’ group (LA, LF, LI, LS and LT) or in the ‘feral’ group (WF and WS; Figure 2A). Furthermore, a large proportion of the SNPs ascertained across the 117 population samples were private to ‘wild’ populations, that is, non‐polymorphic in the ‘feral’ or ‘landrace’ groups (66%, red bar in Figure 2B). By contrast, private SNPs were rare in the ‘landrace’ and ‘feral’ groups (around 7%, represented by the blue and green bars in Figure 2B), and a significant proportion of the polymorphism was shared between them (represented by the purple bar in Figure 2C, accounting for close to 17% of each group's polymorphism). Finally, a substantial fraction of polymorphism was shared by all three groups (in brown, Figure 2C).

### 3.2 PCA on Genotypes

**FIGURE 3:**
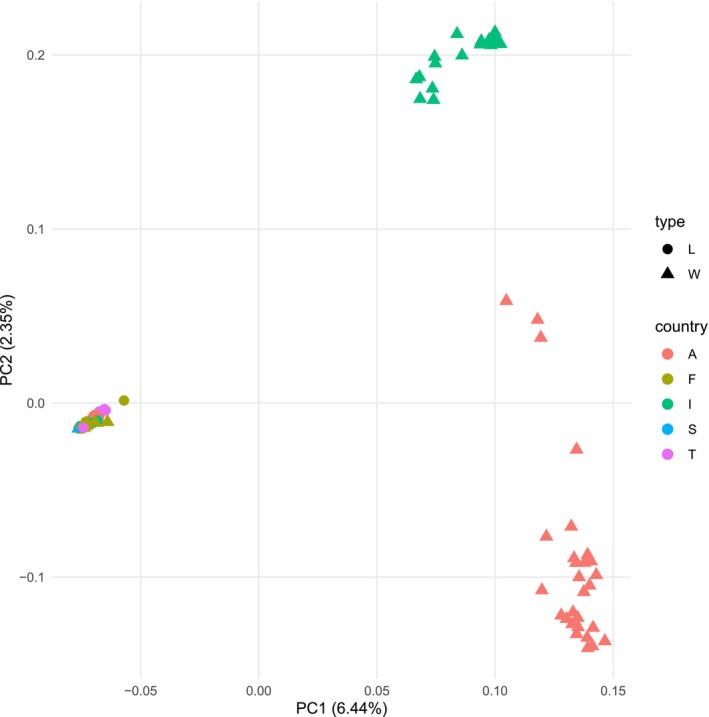
Scatterplot of first two components of the random allele PCA. Circles stand for landraces, while triangles represent spontaneous populations. Colours stand for the countries where the populations were collected: Algeria in red, France in yellow, Italy in green, Slovenia in blue and Tunisia in purple.

Figure 3 shows the scatterplot of the first two components of the random allele PCA. PC1 drastically separated wild populations from Algeria and Italy from a group containing all the landraces as well as the French and Slovenian spontaneous populations, and explained 6.44% of the variation. PC2 explained 2.35% of the allelic variation and separated the wild populations from Algeria from the wild populations from Italy. None of the first six main components managed to separate the French spontaneous populations from the cluster of the landraces (data not shown). The results of the DAPC (Text S1) confirm this finding, identifying six genetic clusters. One cluster is specific to Italian wild populations, while another is specific to Algerian wild populations. The French and Slovenian spontaneous populations are split between two other clusters, with each cluster also including landraces from France, Algeria, Tunisia and Slovenia (Figure 1 in Text S1).

The genetic structure revealed by the PCA was also evident in the analysis of pairwise $F_{\mathrm{ST}}$, which clearly separated the Algerian and Italian wild populations from all landraces and feral populations. The $F_{\mathrm{ST}}$ analysis also identified three outlier populations (the French landrace SAVI and the two Italian landraces PETT_J and PETT_K), which were highly differentiated from all the others. Overall, the $F_{\mathrm{ST}}$ values tended to be remarkably high (Figure S1).

### 3.3 Origin of the Spontaneous Populations: *F*‐Statistics and Admixture

A test for significantly negative $F_{3}$ statistics was performed on all population trios. Among the whole set of trios (*n* = 780,390), only six were found significant at the 95% threshold (five Algerian landraces and one spontaneous Italian population), and none of them involved any of the French or Slovenian spontaneous populations as the admixed one (Table 1).

**TABLE 1:** Summary of the admixture tests for the five Algerian landraces and the Italian spontaneous population that showed significant evidence for admixture. For each admixed population, we show only the source populations associated with the smallest Z‐score. The population names are encoded as species_country_locality_type_replicate where species is BR for *Brassica rapa* ; country is A for Algeria, F for France, I for Italy, S for Slovenia and T for Tunisia; and type is L for Landraces and W for spontaneous.

Admixed population	Source populations	*F*_3_	Z‐score
BR_A_HAYO_L_A	BR_A_TIZI_L_A, BR_F_MONB_W_A	−0.0000142	−1.98
BR_A_MECH_L_A	BR_T_CHEN_L_A, BR_T_BEJA_L_A	−0.0001246	−3.13
BR_A_RMAD_L_A	BR_F_COMB_L_A, BR_T_CHEN_L_A	−0.0002002	−2.31
BR_A_MEGH_L_A	BR_F_COMB_L_A, BR_T_CHEN_L_A	−0.0002620	−3.15
BR_A_AOUL_L_A	BR_A_TIMI_L_A, BR_A_TSAB_L_B	−0.0002833	−10.68
BR_I_POLL_W_A	BR_I_ISNE_W_A, BR_T_MOKN_L_A	−0.0005438	−3.16

**FIGURE 4:**
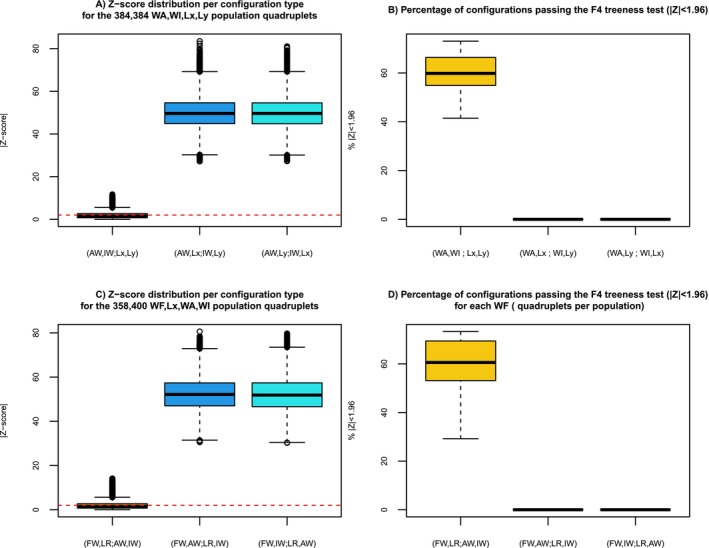
Test showing that the spontaneous populations in Algeria (WA) and Italy (WI) are closer to each other than to any landrace (Lx and Ly), with the boxplot of the |Z‐scores| for the three possible configurations including one WI, one WA and two landraces (A) and the results of the tests of treeness (B). Test showing that the French spontaneous populations (WF) are closer to any landrace than to the Algerian and Italian spontaneous populations, with the boxplot of the |*Z*‐scores| for the three possible configurations including one WF, one WA, one WI and one landrace (Lx) (C) and the results of the tests of treeness (D).

After excluding the six populations showing evidence of admixture (Table 1), we used $F_{4}$‐based treeness tests to explore the proximity between spontaneous populations and landraces (Text S2). First, we focused on Algeria and Italy by creating all possible population quadruplets including one WI, one WA and two landraces (Lx and Ly). For each of the 384,384 quadruplets, the tree configuration associating WA and WI (WA, WI; Lx, Ly) always displayed the lowest |*Z*‐score| compared to the two alternative configurations, even with landraces from Algeria or Italy (Figure 4A). In addition, these other configurations never passed the treeness test, while the (WA, WI; Lx, Ly) configuration passed the treeness test in a large proportion of quadruplets where they were involved (42.3%–69.7%, Figure 4B). This confirms that the spontaneous populations in Algeria and Italy are closer to each other than to any landrace, as expected if they are truly wild.

Secondly, we focused on the French spontaneous populations WF and created the all the quadruplets including one WF population, one spontaneous population from Algeria (WA) and one from Italy (WI), as well as one landrace (Lx). For the 358,400 quadruplets, the tree configuration associating WF and Lx (WF, Lx; WA, WI) consistently showed the lowest |*Z*‐score| (Figure 4C). The two alternative configurations never passed the treeness test (i.e., $\left|Z\right|\gg 1.96$), while the (WF, Lx; WA, WI) configuration passed the treeness test in a large proportion of quadruplets (29.2%–73.3%; Figure 4D). Together, these results strongly suggest that the WF populations are more closely related to other landraces than to any other wild populations. This is consistent with two alternative hypotheses: either the WF populations are the closest wild ancestors of all the landraces, or they are feral (and derived from the French landraces) rather than truly wild.

**FIGURE 5:**
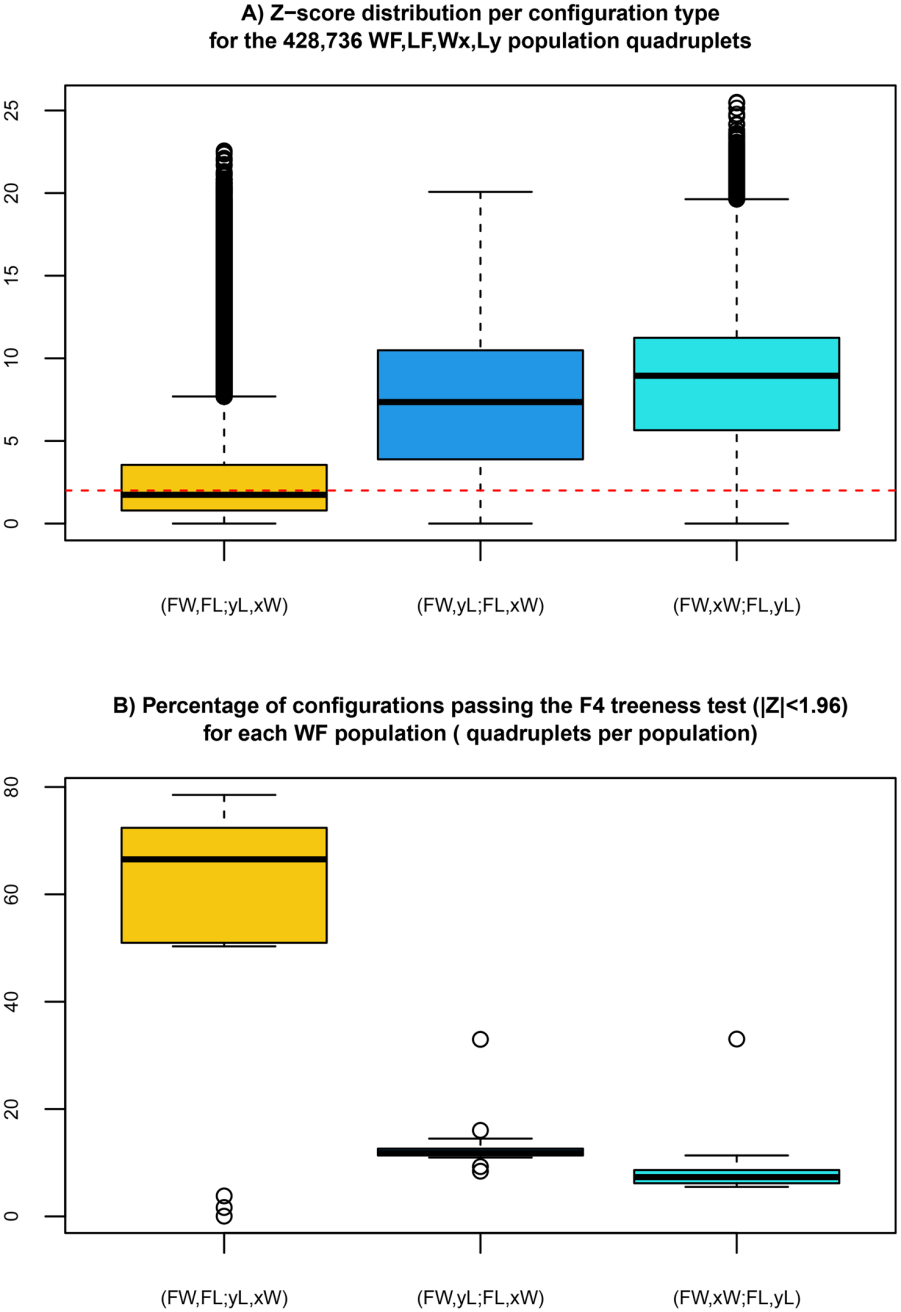
Test showing that the French spontaneous populations (WF) are closer to French landraces (LF) than to other spontaneous populations (from Algeria or Italy, Wx) or to other landraces (Ly), with the boxplot of the |*Z*‐scores| for the three possible configurations including one WF, one LF, one spontaneous population and one landrace (A) and the results of the tests of treeness (B).

In order to differentiate between these two hypotheses, we considered all 428,736 possible combinations of a WF population, a wild population (Wx) from Algeria (x = A) or Italy (x = I), a French landrace (LF), and a landrace from another country (Ly). As expected under the ferality hypothesis, configurations associating WF and LF tended to display the lowest |*Z*‐score| (a large proportion of which passed the treeness test; Figure 5A). However, contrary to previous tests, the two alternative configurations sometimes passed the treeness test (Figure 5B). This is primarily due to three WF populations that rarely pass the treeness test for the (WF, LF; Ly, Wx) configuration. Two of these populations (BR_F_FOUG_W_A from Fougeré in Vendée and BR_F_HILA_W_A from Saint‐Sève in Gironde) seem to be closer to an Algerian or a Tunisian landrace ((WF, Ly; LF, Wx) configuration) whereas the third one (BR_F_COLO_W_A from Saint‐Colomé in the Pyrénées‐Atlantiques, the southernmost sample site in France) is an exception as it seems closer to an Italian spontaneous population ((WF, Wx; LF, Ly) configuration). Overall, most of the WF spontaneous populations are closest to French landraces, providing strong support for the ferality hypothesis.

Similarly, we analysed the Slovenian spontaneous populations by forming the 22,400 quadruplets involving one WS, one WA and one WI, as well as any landrace Lx. As for French spontaneous populations, the lowest |*Z*‐score| was consistently observed for the tree configuration associating WS and Lx (WS, Lx; WA, WI; Figure S2A) and this configuration was the only one passing the treeness test (Figure S2B). This suggests that, as with the French spontaneous populations, the Slovenian population is more closely related to the landraces than to any other wild population (Figure S2C). In addition, the configuration that groups WS with a Slovenian landrace is generally the one with the lowest |*Z*‐score| and passes the treeness test (Figure S2D). When the Slovenian landrace is replaced by another landrace, the test is also successful.

Finally, we identified the closest landrace proxy for each feral population ${\mathrm{WF}}_{t}$ (i.e., the 17 French and Slovenian spontaneous populations) as the one that most frequently maximized the $F_{3}\left(\mathrm{Wx};{\mathrm{WF}}_{t},\mathrm{Ly}\right)$ when considering each of the potential wild ancestors (out of the 44 WI and WA). In 16 out of 17 cases, the most likely ancestor of the feral population was a French landrace (Table 2).

**TABLE 2:** Closest landrace proxy for each feral population ${\mathrm{WF}}_{t}$ (i.e., the 17 French and Slovenian spontaneous populations), determined as the one that most frequently maximized the $F_{3}\left(\mathrm{Wx};{\mathrm{WF}}_{t},\mathrm{Ly}\right)$ when considering each of the potential wild ancestors (out of the 44 WI and WA).

Feral population	Proxy of the source population	Number of occurrences
BR_F_RENN_W_A	BR_F_GACI_L_A	44
BR_F_MORD_W_A	BR_F_GACI_L_A	29
BR_F_FOUG_W_A	BR_F_COMB_L_A	40
BR_F_BRUZ_W_A	BR_F_GACI_L_A	44
BR_F_CATH_W_A	BR_F_GACI_L_A	42
BR_F_LOUB_W_A	BR_F_AUGN_L_A	42
BR_F_LOUP_W_A	BR_S_LJUB_W_F	30
BR_F_MONB_W_A	BR_F_AMBE_L_A	24
BR_F_RAUZ_W_A	BR_F_AMBE_L_A	28
BR_F_PLEB_W_A	BR_F_GACI_L_A	44
BR_F_CHAV_W_A	BR_F_GACI_L_A	44
BR_F_FLAU_W_A	BR_F_AMBE_L_A	31
BR_F_HILA_W_A	BR_F_MERI_L_A	32
BR_F_SAUV_W_A	BR_F_AUGN_L_A	36
BR_F_PELL_W_A	BR_F_ESCH_L_A	17
BR_F_COLO_W_A	BR_F_COMB_L_A	32
BR_S_LJUB_W_F	BR_F_GEOR_L_A	42

### 3.4 Morphological Analysis

#### 3.4.1 Analysis of Root Shape and Colour

The three qualitative variables (root shape, root external and internal colours) were analysed using a MCA (Figure S3). The first axis explained 12.3% of the variance and separated the “golden turnips” (round shape and yellow colour external and internal) from the other root phenotypes. The 2nd axis captured 8.7% of the variance and separated roots with an elliptical shape and green colour from non‐swollen or triangular white roots. All the landraces were highly variable for these two dimensions, unlike the Italian and Algerian wild populations, that all clustered together as a group of non‐swollen white roots (Figure S3). On this space, the French and Slovenian spontaneous populations were positioned close to the Algerian and Italian wild populations, but showed slightly more variance.

#### 3.4.2 Analysis of Quantitative Variables

**FIGURE 6:**
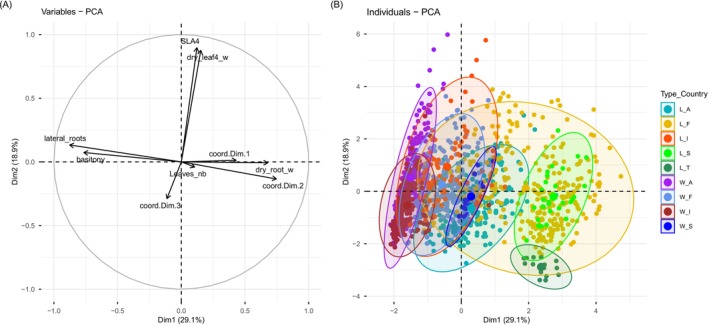
Principal Component Analysis on quantitative morphological variables, including the coordinates on the first three axes of the MCA on categorical variables describing the root shape and colour (coord.Dim.1, coord.Dim.2 and coord.Dim.3). (A) Correlation circle from principal component analysis; (B) plot of the individual coordinates on the axes 1 and 2, where colours stand for the type of population (L_ for landraces and W_ for spontaneous populations) combined with the country of origin (A: Algeria, F: France, I: Italy, S: Slovenia, T: Tunisia).

The three first dimensions of the MCA were included in a PCA along with the quantitative (or semi‐quantitative) traits (root and 4th leaf dry weight, specific leaf area of the fourth leaf, number of leaves, quantity of lateral roots and basitony). The first axis of this PCA explained 29.1% of the variance. It contrasted bushy plants with small roots and many lateral roots, negative coordinates on the 2nd axis of the MCA, that is, non‐swollen or triangular white roots, with upright plants bearing few lateral branches and lateral roots, heavier roots and positive coordinates on the 2nd axis of the MCA, that is, elliptical shape and green colour (Figure 6A). All individuals from the Italian and Algerian wild populations clustered together with negative values on this 1st axis, while most individuals from landraces had higher values (Figure 6B). Plants from the French spontaneous populations showed intermediate coordinates. It is interesting to note that the morphological variability was much higher among landraces than among wild populations, as noticed on the MCA plot (Figure S3).

#### 3.4.3 Why Are French Spontaneous Populations Morphologically Intermediate? What Are the Traits That Distinguish Them From Wild Populations?

**FIGURE 7:**
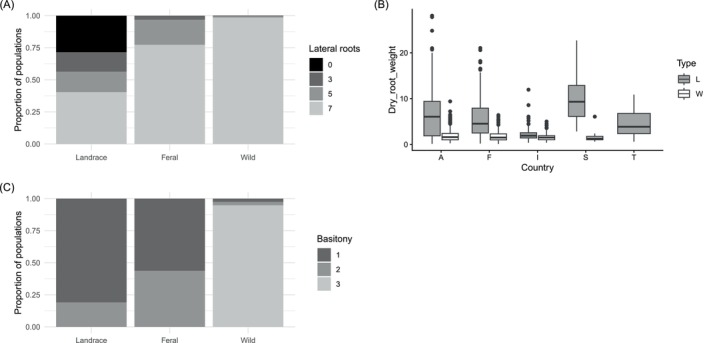
Morphological differences among landraces, spontaneous populations from France and Slovenia (labelled as ‘feral’), and spontaneous populations from Algeria and Italy (labelled as ‘wild’). (A) Number of lateral roots, from 0 = absent to 7 = covering more than half of the root; (B) root dry weight in grams, (C) basitony from 1 = upright port to 3 = bushy.

Spontaneous populations from France and Slovenia were very similar to Algerian and Italian wild populations for root morphology. Both had a high number of lateral roots (*χ* ^2^ = 1.4, *p* = 0.235), significantly higher than in landraces (for wild populations *χ* ^2^ = 25.8, *p* = $10^{-5}$, for French and Slovenian spontaneous populations *χ* ^2^ = 11.4, *p* = 0.009, Figure 7A). All the spontaneous populations were also similar in root dry weight (*p* = 0.766), which was lower than in most landraces by 4.2 g (*p* < 0.0001), even though the landraces were more variable (Figure 7B). We found no effect of the type of land use at the sites where the populations were sampled (detailed in Table S1) on either root dry weight (*p* = 0.630) or lateral roots (*χ* ^2^ = 8.4, *p* = 0.210). Finally, root shape and external colour were also not significantly different between these two groups of spontaneous populations, both having white elongated roots (shape: *χ* ^2^ = 8.6, *p* = 0.035, external colour: *χ* ^2^ = 0.1, *p* = 0.763), unlike landraces that showed more variable shapes and colours (shape: *χ* ^2^ = 15.8, *p* = 0.015, external colour: *χ* ^2^ = 11.7, *p* = 0.008).

In contrast, the above‐ground architecture of plants in the French and Slovenian spontaneous populations resembled that of landraces, with upright growth and few lateral branches, whereas spontaneous plants from Algeria and Italy were bushy (Figure 7C). Pairwise *χ* ^2^ tests confirmed that spontaneous populations from Italy and Algeria had a significantly different basitony compared to landraces (*χ* ^2^ = 75.5, *p* < 10^−16^) and to spontaneous populations from France and Slovenia (*χ* ^2^ = 45.5, *p* < 10^−10^), while the latter did not differ significantly from landraces (*χ* ^2^ = 3.0, *p* = 0.083).

For the other quantitative traits (the fourth leaf dry weight, the specific leaf area of the fourth leaf and the number of leaves), there was no significant difference between the landraces and the wild populations.

#### 3.4.4 Seed Data

**FIGURE 8:**
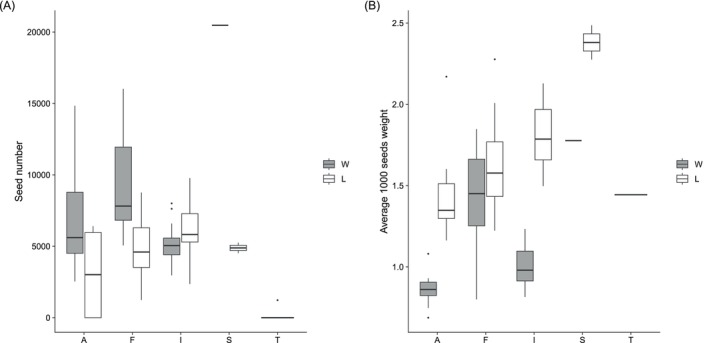
Boxplots for seed number (A) and average thousand seeds weight (B) for landraces and spontaneous populations from Algeria, France, Italy, Slovenia, and Tunisia.

Seed production was assessed using seed number and thousand seeds weight. French and Slovenian spontaneous populations produced the largest number of seeds and differed significantly from landraces (*p* < 0.0001) but not from Algerian and Italian wild populations (*p* = 0.326) (Figure 8A). Landraces produced the heaviest seeds (thousand seeds weight), followed by French and Slovenian spontaneous populations, while Algerian and Italian wild populations exhibited the lightest seeds (Figure 8B). All differences were significant at the 1% *p*‐value threshold.

## 4 Discussion

### 4.1 A Reservoir of Genetic Diversity in Wild Populations of *B. rapa* in Algeria and Italy

In this study we have analysed the genetic diversity, the genetic structure and the morphological characteristics of 117 populations of *Brassica rapa* sampled in the wild or in genebanks of cultivated material from countries located around the Mediterranean basin.

We report high levels of genetic diversity genome‐wide in the wild populations from Algeria and Italy. The diversity described here cannot be compared with previous results reported in the literature because the latter were not measured on populations but on panels of diversity with one accession per country (e.g., nucleotide diversity of 1.10^−5^ (Qi et al. 2017) for a panel including cultivated material). High levels of diversity at the population scale had been described before in Algeria (Aissiou et al. 2018) based on SSR genotyping on nine populations (among which four are also included in this study). This 2018 study was the first to characterize the genetic diversity of wild *B. rapa* accessions from North Africa, and of wild *B. rapa* populations more generally. In Italy, for example, only a few wild accessions had been genotyped until now, for example, one accession from Sicily in Crouch et al. (1995) and one in McAlvay et al. (2021). This last publication suggested that there could be a larger number of truly wild populations of *B. rapa* in Russia, Georgia and Turkey. Yet, there was no information available so far about genetic diversity at the population scale in these regions.

Interestingly, our analysis of 45 wild populations from Italy and Algeria, using up to 30 individuals per population, shows that *B. rapa* natural populations exhibit high genetic diversity. They thus represent an interesting reservoir of genetic diversity to tackle cultivation challenges in this species. The characterization of the genetic diversity of more truly wild populations could have important implications for better understanding the history of domestication of *B. rapa* . Further analyses based on genotypic data of wild populations across a broader geographical range are required in order to identify more precisely the centre of domestication and characterize the reservoir of genetic diversity contained within wild *B. rapa* populations in greater detail.

### 4.2 Multiple Lines of Evidence Support the Feral Origin of Spontaneous *B. rapa* Populations Collected in the Wild in France and Slovenia

Firstly, the genetic diversity of the spontaneous populations sampled in France and Slovenia was lower than that of the wild populations in Algeria and Italy, but it was similar to the genetic diversity of the landraces from France, Algeria, Tunisia, Italy and Slovenia. This reduced genetic diversity can be interpreted as evidence of the impact of the domestication bottleneck. In contrast, spontaneous populations in Italy and Algeria have retained much higher levels of diversity and can therefore be considered as truly wild. The pattern of genetic structure supports this hypothesis, as the French and Slovenian spontaneous populations clustered with the landraces as a whole and were highly differentiated from the wild populations in Algeria and Italy. Finally, our $F_{4}$ analyses of all the population quadruplets showed that, as expected if they were truly wild, the spontaneous populations in Algeria and Italy were closer to each other than to any landrace. By contrast, the French spontaneous populations were more closely related to other landraces than to any other wild populations. This confirms their feral origin. Using $F_{3}$ analyses, we identified the best landrace proxy that could have given rise to each feral population. This analysis pointed towards a French landrace for nearly all of the feral populations. Together with the lack of evidence that the French spontaneous populations could have resulted from admixture events between landraces and wild populations, our results indicate an endoferal origin for these feral populations (Scossa and Fernie 2021; Wu et al. 2021). However, we should note that the configurations associating the Slovenian spontaneous population with a wild one also passed the treeness test a significant number of times (Figure S2D). This may be due to the absence of a close proxy for the landrace ancestral to the Slovenian spontaneous population, or to substantial gene flow from an unsampled wild population. The $F_{3}$ analyses identified a French landrace as the closest proxy to the Slovenian feral population (Table 2). This French landrace is a strain that was brought back from Belgium by a soldier in 1914 and has been passed down through the generations ever since. Nevertheless, this is insufficient evidence to conclude that French or Belgian landraces spread outside the field and became feral in Slovenia. Additional Slovenian landraces are required to determine whether Slovenian feral populations actually derive from French or Belgian landraces, or from other local landraces.

In terms of phenotype, French and Slovenian spontaneous populations were similar to Algerian and Italian wild populations for root morphology (shape and colour but also dry weight and lateral roots) but not for basitony. Overall, they appeared as intermediate in the global phenotype‐based PCA.

These results are consistent with previously published data on *Brassica rapa* , which suggested that most European populations are ferals (Crouch et al. 1995; McAlvay et al. 2021; Saban et al. 2023), while truly wild individuals could be present in Italy, the Caucasus and Siberia (McAlvay et al. 2021). However, this is the first detailed study, based on large population samples, describing the molecular and morphological diversity as well as the genetic structure of wild *B. rapa* populations.

### 4.3 Traits Showing Evidence for Reversion to Pre‐Domestication Traits

While the plants' growth habit of feral populations from France and Slovenia was similar to that of all landraces (basitony), the root morphology was closer to that of Algerian and Italian wild populations in terms of shape and colour, but also dry weight and lateral roots. Given that our genetic results support the feral origin of French and Slovenian spontaneous populations, this result suggests a return towards pre‐domestication traits for root morphology in these populations. This phenotypic change could have been affected by a bottleneck involved in the de‐domestication process, but the low level of diversity in feral populations, similar to landraces, does not favour this hypothesis. Instead, it could be the result of selection if producing a large and colourful root was costly and if this trait was highly polygenic, which would facilitate the response to selection.

Indeed, such traits selected for in landraces could be strongly counter‐selected in the wild. In addition, a high efficiency of the root in resource acquisition, possibly influenced by the presence of lateral roots, is crucial in a wild environment, whereas resource acquisition may be less challenging in the field thanks to irrigation and fertilization (see Figure S4 for images showing the differences in root morphology between landraces, wild and feral populations). We exploited the diversity of land use at the sites where the spontaneous populations were sampled to investigate its potential impact on dry root weight and lateral roots, but found no significant effect. While this is not unexpected given that the populations were all grown in a common environment for one generation prior to the experiment to eliminate any potential maternal effects, it nevertheless shows that there is no evidence of strong selective pressures exerted by the local environment (from cultivated field to semi‐natural areas) on root morphology.

Similarly, for germination traits, a previous study on the same set of populations showed that French *B. rapa* feral populations had lower germination rates and higher germination times compared to landraces (Tiret et al. 2025). The critical factor influencing germination therefore appeared to be whether populations were sampled in the wild or in the field, and not their genetic origin (feral or wild). This suggests that the feral populations tend to revert back to patterns akin to wild populations for germination, probably because the environment is less predictable in the wild than in cultivated fields, so that bet‐hedging strategies (Bewley 1997) based on variable seed dormancy capacities are highly advantageous. Yet, the French spontaneous populations still show little dormancy compared to the Algerian and Italian wild populations, with higher and faster germination (Wagner et al. 2025). This could be because arid Mediterranean conditions select for higher dormancy levels.

Selection towards pre‐domestication traits in feral populations is common, especially for seed dormancy traits (Ayal and Levy 2005; Zhou et al. 2021), or seed dispersal (Wu et al. 2021). However, genomic scans have shown that the genomic pathway to de‐domestication does not necessarily involve reversion of the same alleles targeted by domestication (Mabry, Rowan, et al. 2021). In weedy rice, for example, most accessions carry the domestication allele sh4, which causes delayed shattering in domesticated rice lines, and yet they disperse their seeds at maturity (Li et al. 2017). However, if de‐domestication occurs by back‐mutation to the dominant allele (e.g., the shattering of spiklets in grasses), it can be much faster than domestication by selection of this rare recessive trait (Gressel 2005; Fuller 2007).

### 4.4 Where Do These Feral Populations Come From?

Our results strongly support the hypothesis that the spontaneous populations in France and Slovenia are feral, and raise the question of their origin. One possible scenario is that the feral plants escaped from turnip fields and subsequently lost the ability to produce a tuberous root. This hypothesis is based on the idea of “undoing” domestication, whereby genes revert to a state resembling the original undomesticated state. This argument is similar to the one developed above for germination traits. Alternatively, the feral plants we observed could derive from a *B. rapa* morphotype domesticated for oil that never produced a swollen root. Several varieties of oleiferous forms of *B. rapa* have been cultivated for oil in Europe since the early Middle Ages (turnip rape, *B. rapa ssp oleifera*; Pandolfo et al. 2018). Later, it was progressively replaced by *Brassica napus* and oleiferous forms of *B. rapa* are now mainly cultivated in east Asia (yellow sarson *B. rapa* ssp. *trilocularis* and brown sarson or toria, ssp. *dichotoma*). These oil morphotypes are likely to have been selected independently from the root or vegetable types (Guo et al. 2014), and the turnip rape is genetically close to European turnips (same genetic cluster, McAlvay et al. 2021), which makes it a good candidate for the feral populations in Europe.

Recently, Saban et al. (2023) compared signatures of selection in wild and cultivated *Brassica*. The authors found no overlap in the genomic regions targeted by positive selection between the wild *B. rapa* (from the UK, Turkey, Iran, Sweden, Italy, Egypt and the USA) grouped with the *B. rapa ssp. rapa* (cultivated turnip morphotype) on the one side, and Asian domesticated subspecies (ssp. *chinensis*, *parachinensis*, *pekinensis* and *trilocularis*) on the other side. This suggests that these two groups of populations did not undergo the same selection process. Wild *B. rapa* (ssp. *sylvestris*) and ssp. *rapa* of the turnip morphotype were grouped together because they belonged to the same genetic cluster. The small sample size prevented a direct comparison between wild *B. rapa* (ssp. *sylvestris*) and ssp. *rapa* of the turnip morphotype. This leaves unanswered questions about whether these wild accessions are truly wild or feral, and if feral, which domesticated subspecies they originated from such analyses of signatures of selection could help to identify which cultivated populations are the ancestors of the feral populations; for example, by contrasting the signatures of selection in feral *B. rapa* with either *B. rapa ssp. rapa* (turnip morphotype) or *B. rapa* ssp. *oleifera* (european turnip rape). We expect the crop that is the ancestor of the feral populations to display similar signatures of selection to those seen in the feral populations. Whole‐genome sequencing of weedy rice accessions, alongside the analysis of their phylogenetic proximity to other domesticated and truly wild rice forms, has traced the origin of these invasive forms back to varieties or cultivars that were often grown in the same regions (Qiu et al. 2020). These studies also showed that feral rice has evolved from cultivated rice on multiple occasions at different points in time (Wu et al. 2021), which could also be the case for the widespread *B. rapa* feral populations in Europe and Americas.

### 4.5 Adaptation in Feral Populations?

Although the genetic differentiation between landraces and spontaneous populations from France and Slovenia was small, we cannot rule out the possibility of selective effects affecting feral populations. Selective effects after feralisation can include domestication genes or more broadly domestication traits (e.g., seed dispersal in weedy rice, Li et al. 2017, Gering et al. 2019, Wu et al. 2021) as well as genes underlying adaptation to a new local environment (Burger and Ellstrand 2014; Qiu et al. 2020). This raises the question of testing whether adaptation to the natural environment during feralisation occurs through the fixation of standing variation (albeit limited) or through newly arising mutations (Scossa and Fernie 2021). Additionally, the process of feralisation likely involves bottleneck events before populations can become established outside of cultivated areas, which could have significant implications for genetic load (Gautier et al. 2024). However, genetic load in feral plant populations has received little attention from researchers. Improving our understanding of adaptive diversity and genetic load would enhance the value of feral populations as a genetic resource for plant improvement. Finally, while feral populations can harbour genetic diversity and provide insights into adaptation, they can also present challenges for ecosystem management and agricultural practices. This makes careful monitoring and control strategies essential. For instance, feral populations have the potential to act as vectors for the unintended spread of transgenes into wild relatives. This has already occurred in Argentina, where natural populations of *Brassica rapa* carrying transgenic herbicide resistance have been identified (Pandolfo et al. 2018). The impact of such gene flow could be further increased by the potential for interspecific crosses within the genus Brassica (FitzJohn et al. 2007).

## 5 Conclusion

In this study, we report abundant genetic diversity in a set of 45 wild populations of *Brassica rapa* sampled in Italy and Algeria. These populations form important reservoirs of genetic diversity for *Brassica* crops and require protection as well as further studies, in particular to better describe the geographic range of this wild species. Feral populations of *B. rapa* are frequent and show depleted genetic diversity. Our admixture analyses suggest that they are derived from French landraces, and we found no evidence of gene flow with the wild populations included in our study. Given the wide geographical distribution of feral *B. rapa* populations, further research is needed to understand their history of feralization. This includes how these feral populations spread across Europe and the Americas, potential gene flow with cultivated and wild populations, and putative patterns of local adaptation.

## Author Contributions

A.‐M.C. and J.R. designed the research and acquired the funding. C.F., L.B.‐V., G.D. and A.‐M.C. participated in the collection and the local description of the populations. C.F., L.B.‐V., G.D., M.T. and A.‐M.C. designed and managed the phenotypic experiments. C.F. and G.D. were responsible for preparing the plants for DNA extractions, sampling leaf tissue and extracting DNA in pools. S.G., S.B. and M.G. performed the SNP calling analyses and contributed to several analyses of diversity and admixture. J.R. analysed the population structure and designed the core collection. L.G. performed the analyses and wrote the manuscript with the help of J. Ronfort. S.B., L.B.‐V., A.‐M.C., C.F., M.G., M.T. and S.B. contributed to writing and editing the manuscript.

## Funding

The investigation was supported by EU's Horizon 2020 Prima funding (grant no. 1425), entitled “BrasExplor: Wide exploration of genetic diversity in Brassica species for sustainable crop production”; by INRAE through the TSARA initiative (Transforming food systems and agriculture through a partnership research with Africa), which fosters Franco‐Algerian collaborations; and by the France Génomique National infrastructure, funded as part of the “Investissements d'Avenir” program managed by the Agence Nationale pour la Recherche (contract ANR‐10‐INBS‐09).

## Conflicts of Interest

The authors declare no conflicts of interest.

## Supporting information


**Text S1:** Building core‐collections of *Brassica rapa* using pool‐seq data: three nested core‐collections composed of 12, 24 and 48 populations—denoted respectively as CC12, CC24, and CC48—were assembled to represent the bulk of the diversity contained in the complete collection of *Brassica rapa* populations.

**Text S2:** Rationale of the 4‐populations test of treeness.

**Figure S1:** Heatmap representing the genome‐wide estimates of $F_{\mathrm{ST}}$ coefficients for each of the population pairs, estimated using the compute.fstat() function in the R package poolfstat. The populations names are encoded as species_country_locality_type_replicate where species is BR for *Brassica rapa* ; country is A for Algeria, F for France, I for Italy, S for Slovenia and T for Tunisia; and type is L for Landraces and W for spontaneous.
**Figure S2:** Test showing that the Slovenian spontaneous population (WS) is closer to any landrace (Lx) than to the Algerian and Italian spontaneous populations (WA and WI), with the boxplot of the |Z‐scores| for the three possible configurations including one WS, one WA, one WI and one landrace (Lx) (A) and the results of the tests of treeness (B). Test showing that the Slovenian spontaneous populations (WS) tend to be closer to Slovenian landraces (LS) or other landraces (Ly) than to other spontaneous populations (from Algeria or Italy, Wx), with the boxplot of the |Z‐scores| for the three possible configurations including one WS, one LS, one spontaneous population and one landrace (C) and the results of the tests of treeness (D).
**Figure S3:** Plot of the Multiple Correspondence Analysis on qualitative morphological variables describing the root shape and colour (internal and external), showing individual coordinates on the dimensions 1 and 2 (A) and 3 and 2 (B). The codes for the quantitative variables used (root colour and shape) are detailed by the International Board for Plant Genetic Resources (IBPGR and CEC 1990).
**Figure S4:** Comparison of root morphology in landraces (left column), feral populations (centre) and wild populations (right) from several countries. Each panel shows a photograph of the root (whole and cut) of one plant, which was collected from the common garden in Rennes. The names of the populations are written on the white labels.

**Table S1:** Type of land use for the spontaneous populations sampled in the wild in Algeria, France, Italy and Slovenia. The land use categories are arranged along a gradient of agricultural intensity ranging from cultivated fields or annual or perennial crops to semi‐natural or urban areas.

## Data Availability

The short‐read resequencing data are available on NCBI's sequence read archive (SRA) under the project PRJNA1174687. The VCF file has been submitted to Zenodo (DOI: 10.5281/zenodo.18329476) and can be accessed via the following link: https://zenodo.org/records/18329477. Phenotypic data along with the scripts used for the phenotypic and genetic analyses are available on the INRAE dataportal (DOI: 10.57745/CYGTND) and can be accessed via the following link: https://entrepot.recherche.data.gouv.fr/dataset.xhtml?persistentId=doi:10.57745/CYGTND. Seeds of the accessions are available at the following Genetic Resource Centres: BrACySol for French accessions and Agricultural Institute of Slovenia for Slovenian accessions. For inquiries regarding Algerian, Italian, and Tunisian accessions, please refer to the contact specified in Table S3 in Tiret et al. (2025).
