# Supplementary material for: Genetic and Morphological Diversity in Spontaneous Populations of Brassica rapa: How Do Feral Populations Differ From Wild Ones?
Source: Mol Ecol. 2026 Jul 8;35(13):e70461. doi: 10.1111/mec.70461 (PMC13346339; doi:10.1111/mec.70461)
Supplement: Supplementary file 1 — Text S1: Building core‐collections of Brassica rapa using pool‐seq data: three nested core‐collections composed of 12, 24 and 48 populations—denoted respectively as CC12, CC24, and CC48—were assembled to represent the bulk of the diversity contained in the complete collection of Brassica rapa populations. [file MEC-35-e70461-s001.docx]

**Supplementary Text 1. Building core-collections of *Brassica rapa* using pool-seq data.**

Three nested core-collections composed of 12, 24 and 48 populations - denoted respectively as CC12, CC24, and CC48 - were assembled to represent the bulk of the diversity contained in the complete collection of *Brassica rapa* populations. We used pool-seq data and population genetic structure of the complete collection as described below.

**1. Pool-sequencing and population structure analysis**:

Let’s remember that the complete collection included 62 spontaneous populations collected in Algeria (28), Italy (17), France (16) and Slovenia (1), and 55 *B. rapa* landraces collected either in farms in Algeria (22), Tunisia (5) and Italy (5) or from Biological Resource Centers (BRC) maintaining old landraces in France (BRC BrACySol, 21) and Slovenia (BRC KIS, 2). The majority of the local landraces collected were turnips (subsp. *rapa*) with the exception of few broccoletto (subsp. *sylvestris* var. *esculenta*) selected by Italian farmers.

For each population, DNA bulks were built from 30 plants, representing 11-30 mother plants collected in situ, and sequenced with Illumina Hiseq technology, providing a genome-wide picture of the within population nucleotide diversity. We used a subset of randomly chosen SNPs in intergenic regions (n = 13675) and performed a ‘random allele’ Principal Component Analysis (PCA) and a discriminant analysis of principal components, DaPC (Jombart et al. 2010) implemented in the R-package adegenet to infer the population genetic structure of the collection. The DaPC method identified K = 6 genetic clusters and Figure 1 describes their composition and diversity.

| **Figure 1.** (A) Distribution of the 117 B. rapa populations across the 6 genetic clusters according to their country of origin (A: Algeria, F: France, I: Italy, S: Slovenia, T: Tunesia) and type, i.e., cultivated (L for Landraces) versus spontaneous (W for Wild) populations. (B) General description of the 6 clusters: number of populations and diversity as measured by the number of polymorphic sites per cluster (out of 13675 SNP analysed). | 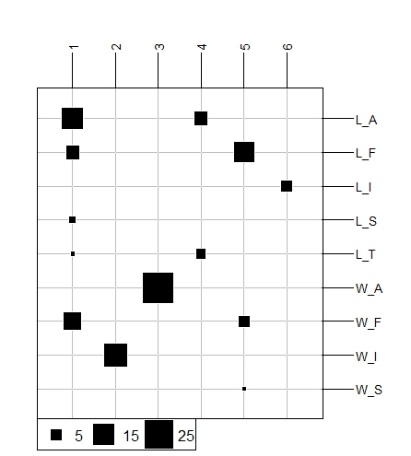  (A) |
| --- | --- |
| (B)   \| **Clusters** \| **1** \| **2** \| **3** \| **4** \| **5** \| **6** \| \| --- \| --- \| --- \| --- \| --- \| --- \| --- \| \| **General description** \| Landraces France, Algeria, Slovenia, Tunesia + Spontaneous France \| Spontaneous Italy \| Spontaneous algeria \| landraces Algeria + Tunesia \| Landraces France + Spontaneous France and Slovenia \| Landraces Italy \| \| **Number of populations** \| 38 \| 17 \| 27 \| 11 \| 20 \| 5 \| \| **Number of polymorphic sites overall in cluster** \| 10310 \| 8906 \| 8152 \| 8596 \| 9662 \| 8267 \| | |

**2. Construction of nested core-collections**

Core-collections were established by stratified sampling from the 6 genetic groups described above, using the following method. As suggested by Franco *et al*. (2005), the number of populations we sampled per cluster was set to be proportional to the within-cluster diversity, using as weight value the number of polymorphic sites observed for a randomly selected set of 13 675 SNPs spanning the genome. Table 1 gives the resulting number of populations representing each cluster in the tree core-collections.

| Core-collection | Cluster 1 | Cluster 2 | Cluster 3 | Cluster 4 | Cluster 5 | Cluster 6 |
| --- | --- | --- | --- | --- | --- | --- |
| CC12 - *n* = 12 | 2 | 2 | 2 | 2 | 2 | 2 |
| CC24 - *n* = 24 | 5 | 4 | 4 | 4 | 5 | 2 |
| CC48 - *n* = 48 | 10 | 9 | 8 | 9 | 10 | 2 |

**Table 1.** Number of populations representing each cluster in the 3 assembled core-collections of *Brassica rapa*.

The selection of populations within each cluster was then carried out as follows: we selected the population with the highest number of polymorphic sites, then, the second and subsequent populations were chosen in order to maximize the number of polymorphic sites of the core-collection. The core-collections were nested so the CC24 and CC48 were constrained to include the populations already comprised in CC12 and CC24 respectively. Figure 2 shows the geographic distribution of the chosen populations in the three core-collections. See Table 2 for a detailed presentation of the nested core-collections.

Seeds are available on request in the different partner countries (e.g. Biological Resource Centers (BRC) for France <https://eng-igepp.rennes.hub.inrae.fr/about-igepp/platforms/bracysol> and Slovenia (https://www.kis.si/en/).

**Figure 2.** Geographic distribution of the populations included in the nested core-collections of *B.rapa*.


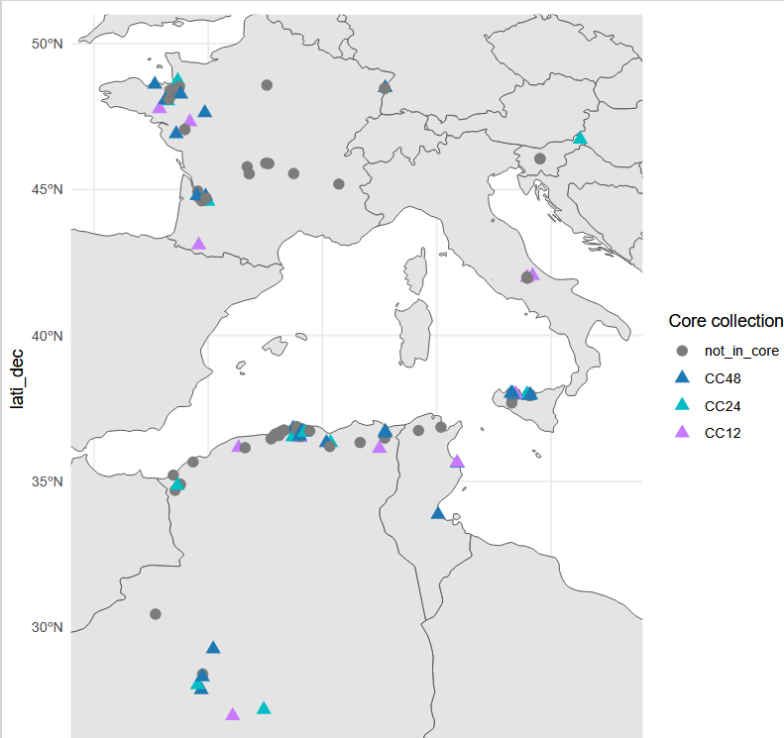


| **PopID** | **Core collection** | **Genetic cluster** | **Wild versus Landrace** | **Morphotype** | **Collection site** | **Country** |
| --- | --- | --- | --- | --- | --- | --- |
| BR-A-AOUL-L-A | CC12 | 4 | L | Turnip | Aoulef-Tidikelt | Algeria |
| BR-A-HAYO-L-A | CC12 | 1 | L | Turnip | Assi-Youcef | Algeria |
| BR-F-GACI-L-A | CC12 | 5 | L | Turnip | Gacilly | France |
| BR-F-LAUR-L-A | CC12 | 5 | L | Turnip | St Laurent de la plaine | France |
| BR-I-LAMA-L-A | CC12 | 6 | L | Turnip | Lama dei Peligni | Italy |
| BR-I-PETT-L-H | CC12 | 6 | L | Turnip | Pettoranosul Gizio | Italy |
| BR-T-MONA-L-A | CC12 | 4 | L | Turnip | Sahel-Monastir | Tunisia |
| BR-A-CHLE-W-A | CC12 | 3 | W | WildTurnip | Chlef | Algeria |
| BR-A-GUEL-W-A | CC12 | 3 | W | WildTurnip | Guelma | Algeria |
| BR-F-COLO-W-A | CC12 | 1 | W | WildTurnip | Saint-Colome | France |
| BR-I-GRAT-W-B | CC12 | 2 | W | WildTurnip | Gratteri | Italy |
| BR-I-MISI-W-B | CC12 | 2 | W | WildTurnip | Misilmeri | Italy |
| BR-A-INSA-L-B | CC24 | 4 | L | Turnip | InSallah | Algeria |
| BR-A-MEGH-L-A | CC24 | 1 | L | Turnip | Meghras | Algeria |
| BR-A-TOUA-L-A | CC24 | 4 | L | Turnip | Touat (Bouda) | Algeria |
| BR-A-TZRA-L-A | CC24 | 1 | L | Turnip | Tizi-Rached | Algeria |
| BR-A-KADI-W-A | CC24 | 3 | W | WildTurnip | Kadiria | Algeria |
| BR-A-TLMC-W-A | CC24 | 3 | W | WildTurnip | Tlemcen | Algeria |
| BR-F-BRUZ-W-A | CC24 | 5 | W | WildTurnip | Bruz | France |
| BR-F-CHAV-W-A | CC24 | 5 | W | WildTurnip | Chavoy | France |
| BR-F-HILA-W-A | CC24 | 1 | W | WildTurnip | Saint-Sève | France |
| BR-I-CAST-W-B | CC24 | 2 | W | WildTurnip | Castelbuono | Italy |
| BR-I-LASC-W-A | CC24 | 2 | W | WildTurnip | Lascari | Italy |
| BR-S-LJUB-W-F | CC24 | 5 | W | WildTurnip | Selo | Slovenia |
| BR-A-ADRA-L-A | CC48 | 4 | L | Turnip | Adrar | Algeria |
| BR-A-RMAD-L-A | CC48 | 1 | L | Turnip | Remadna | Algeria |
| BR-A-TIMI-L-A | CC48 | 4 | L | Turnip | Timimoun-Gourara | Algeria |
| BR-A-TIZI-L-A | CC48 | 1 | L | Turnip | Tizi-Ouzou | Algeria |
| BR-A-TSAB-L-B | CC48 | 4 | L | Turnip | Tsabit-AdrarB | Algeria |
| BR-F-ESCH-L-A | CC48 | 5 | L | Turnip | Eschau | France |
| BR-F-HERB-L-A | CC48 | 5 | L | Turnip | Hebergement | France |
| BR-F-PARC-L-A | CC48 | 5 | L | Turnip | Parce | France |
| BR-T-CHEN-L-A | CC48 | 4 | L | Turnip | Chenini-Gabes | Tunisia |
| BR-T-MOKN-L-A | CC48 | 4 | L | Turnip | Moknine | Tunisia |
| BR-A-ANAB-W-A | CC48 | 3 | W | WildTurnip | Annaba | Algeria |
| BR-A-CAPD-W-A | CC48 | 3 | W | WildTurnip | CapDjinet | Algeria |
| BR-A-DREA-W-A | CC48 | 3 | W | WildTurnip | Drean | Algeria |
| BR-A-MECH-W-A | CC48 | 3 | W | WildTurnip | Mechtras | Algeria |
| BR-F-FOUG-W-A | CC48 | 1 | W | WildTurnip | Fougeré | France |
| BR-F-MONB-W-A | CC48 | 1 | W | WildTurnip | Monbazillac | France |
| BR-F-MORD-W-A | CC48 | 5 | W | WildTurnip | Mordelles | France |
| BR-F-PLEB-W-A | CC48 | 5 | W | WildTurnip | Pléboulle | France |
| BR-F-RAUZ-W-A | CC48 | 1 | W | WildTurnip | Rauzan | France |
| BR-I-ISNE-W-B | CC48 | 2 | W | WildTurnip | Isnello | Italy |
| BR-I-MONR-W-A | CC48 | 2 | W | WildTurnip | Monreale | Italy |
| BR-I-PIAN-W-A | CC48 | 2 | W | WildTurnip | Pianadegli Albanesi | Italy |
| BR-I-POLL-W-A | CC48 | 2 | W | WildTurnip | Pollina | Italy |
| BR-I-MARI-W-A | CC48 | 2 | W | WildTurnip | Marineo | Italy |

**Table 2.** Detailed composition of *Brassica rapa* nested Core-Collections (CC12, CC24 and CC48).

**References**

Brown A. 1989. Core collections: a practical approach to genetic resources management. Genome, 31:818–24.

Franco J, Crossa J, Taba S, Shands H. 2005. A sampling strategy for conserving genetic diversity when forming core subsets. Crop Sci. 45: 1035–44.

Jombart T, Devillard S, Balloux F (2010) Discriminant analysis of principal components: a new method for the analysis of genetically structured populations. BMC Genetics, 11, 94.
