# Supplementary material for: Genetic and Morphological Diversity in Spontaneous Populations of Brassica rapa: How Do Feral Populations Differ From Wild Ones?
Source: Mol Ecol. 2026 Jul 8;35(13):e70461. doi: 10.1111/mec.70461 (PMC13346339; doi:10.1111/mec.70461)
Supplement: Supplementary file 2 — Text S2: Rationale of the 4‐populations test of treeness. [file MEC-35-e70461-s002.docx]

**Supplementary Text 2. Rationale of the 4-populations test of treeness**

**1. Basic idea (see Patterson et al. 2012)**

For a given quadruplet of populations A, B, C and D, the $\boldsymbol{F}_{\boldsymbol{4}}$ **statistic** is defined as:

$$F_{4}\left( A,B;C,D \right)\mathbb{=E}\left[ \left( p_{A}-p_{B} \right)\left( p_{C}-p_{D} \right) \right]$$

where $p_{X}$ is the allele frequency in population $X$.

Under a **pure tree** (no admixture), one of the three possible unrooted topologies (namely (A,B;C,D); (A,C;B,D) or (A,D;B,C)) should yield $\mathbb{E}\left[ F_{4} \right]=0$, while the other two are expected to be positive (reflecting shared drift). For example, if the true topology is (A,B;C,D), then $F_{4}\left( A,B;C,D \right)\approx0$, while $F_{4}\left( A,C;B,D \right)$ and $F_{4}\left( A,D;B,C \right)$ reflect drift along internal branches.

Hence for a given quadruplets of populations, one may compute the $F_{4}$ associated with each of the three possible unrooted topology. If exactly one $F_{4}$ per quartet is ~0 (i.e., one quadruplet configuration passes the treeness test), the quartet is consistent with a tree defined by the corresponding unrooted topology. Conversely, if *all three* are significantly nonzero, the populations cannot be fit by a tree, which implies **admixture or gene flow** in their shared history.

In other words, the intuition behind the treeness test is to ask the question: are allele frequency differences between one pair of populations **correlated** with those between another pair?

- Under a tree: independent drift paths → no correlation → $F_{4}=0$
- Nonzero (F_4): overlapping drift paths → signal of **admixture or shared ancestry** not captured by a tree.

**2. Implementation in poolfstat**

Steps followed in the package (see vignette):

1. Compute $F_{4}$ for all population quadruplets. For a given quadruplet of populations A, B, C and D, there are three possible configurations (i.e., (A,B;C,D); (A,C;B,D) and (A,D;B,C)) after excluding the configurations obtained by permuting populations within pairs (see section 4.1 of the poolfstat vignette, Gautier et al. 2022). Indeed, when comparing two pairs of populations (A,B) and (C,D), the $F_{4}$ statistics for the eight quadruplets (A,B;C,D); (B,A;C,D); (A,B;D,C); (B,A;D,C); (C,D;A,B); (C,D;B,A); (D,C;A,B) and (D,C;B,A) have the same absolute value by definition of the $F_{4}$ parameter:

$$F_{4}\left( A,B;C,D \right)=F_{4}\left( B,A;D,C \right)=F_{4}\left( C,D;A,B \right)=F_{4}\left( D,C;B,A \right)$$

$${- F}_{4}\left( A,B;C,D \right)=F_{4}\left( B,A;C,D \right)=F_{4}\left( C,D;B,A \right)=F_{4}\left( D,C;A,B \right)=F_{4}\left( A,B;D,C \right)$$

In poolfstat, if $i_{P}$ is the index of population $P$ in the *popnames* or *poolnames* slots of the *countdata* or *pooldata* objects (i.e., the column order in the corresponding allele or read count data matrices) used to obtain the *fstats* object, the $F_{4}\left( A,B;C,D \right)$ configurations reported in the slot ***f4.values*** (and ***Dstat.values***) satisfy $i_{A}<i_{B}$; $i_{A}<i_{C}$ and $i_{C}<i_{D}$.

1. Estimate standard errors with block-jackknife.
2. Compute **Z-scores**:

$$Z= \frac{\hat{F}_{4}}{s.e.(\hat{F}_{4})}$$

3. Apply a significance threshold (e.g. $\left| Z \right|<1.96$ for 95% confidence.
4. Retain only population sets where **all quadruplets** satisfy treeness (i.e. only one topology per quadruplet has $\left| Z \right|$ below threshold).

**3. Identifying the closest landrace proxy for each feral population**

If there is no admixture, the expected value of an f-statistic can be computed from the overlap of the two drift paths in the single phylogenetic tree relating the populations (Patterson et al. 2012). In figure 1A, $F_{3}\left( Wx ; {WF}_{t},Ly \right)$ measures the overlap between the genetic drift paths $Wx$ → ${WF}_{t}$ and $Wx$ → $L_{1}$, and is represented by the purple path.

| 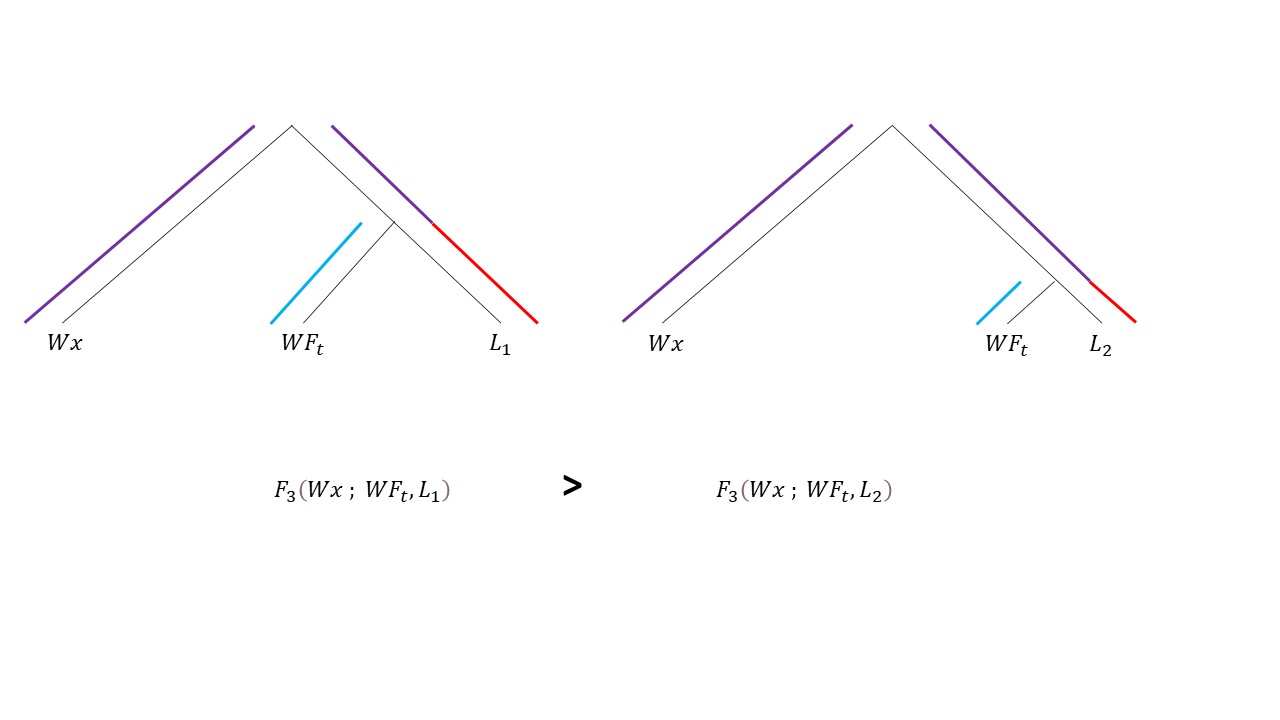 | 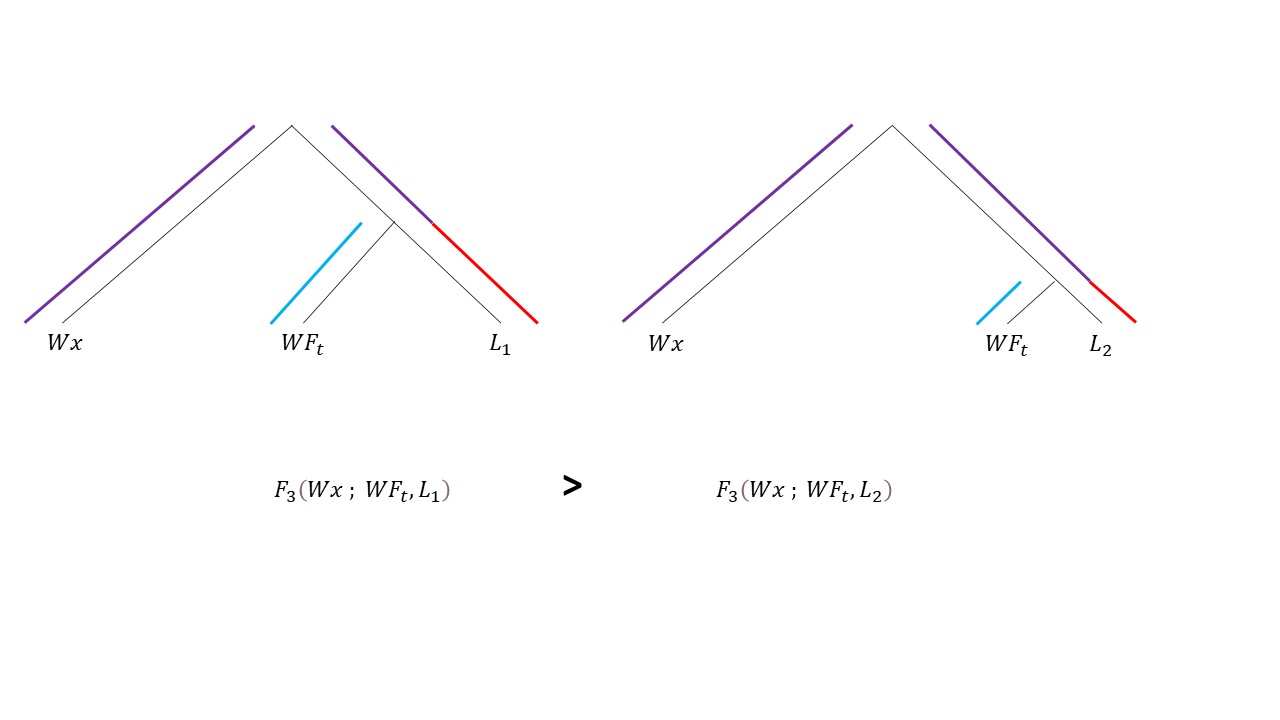 |
| --- | --- |
| **Figure 1A.** Visual representation of the f-statistic in a single phylogenetic tree relating the populations if there is no admixture. $F_{3}\left( Wx ; {WF}_{t},Ly \right)$ can be computed from the overlap of the two drift paths, i.e. the purple path. | **Figure 1B.** If ${WF}_{t}$ and $L_{2}$ are more closely related, the overlap between the two drift paths is longer, which translates into a larger value for $F_{3}\left( Wx ; {WF}_{t},L_{2} \right)$. |

For a given wild ancestor $Wx$, the closest landrace proxy of the feral population ${WF}_{t}$ may thus be considered as the one maximizing $F_{3}\left( Wx ; {WF}_{t},Ly \right)$.

**References**

Gautier, M., R. Vitalis, L. Flori, and A. Estoup. 2022. f-Statistics estimation and admixture graph construction with Pool-Seq or allele count data using the R package poolfstat. Mol Ecol Resour **22**:1394-1416.

Patterson, N., P. Moorjani, Y. Luo, S. Mallick, N. Rohland, Y. Zhan, T. Genschoreck, T. Webster, and D. Reich. 2012. Ancient admixture in human history. Genetics **192**:1065-1093.
