# Supplementary material for: Genetic and Morphological Diversity in Spontaneous Populations of Brassica rapa: How Do Feral Populations Differ From Wild Ones?
Source: Mol Ecol. 2026 Jul 8;35(13):e70461. doi: 10.1111/mec.70461 (PMC13346339; doi:10.1111/mec.70461)

Supplementary Figures

Supplementary Figure S1. Heatmap representing the genome-wide estimates of F_ST coefficients for each of the population pairs, estimated using the compute.fstat() function in the R package poolfstat. The populations names are encoded as species_country_locality_type_replicate where species is BR for Brassica rapa; country is A for Algeria, F for France, I for Italy, S for Slovenia and T for Tunisia; and type is L for Landraces and W for spontaneous.

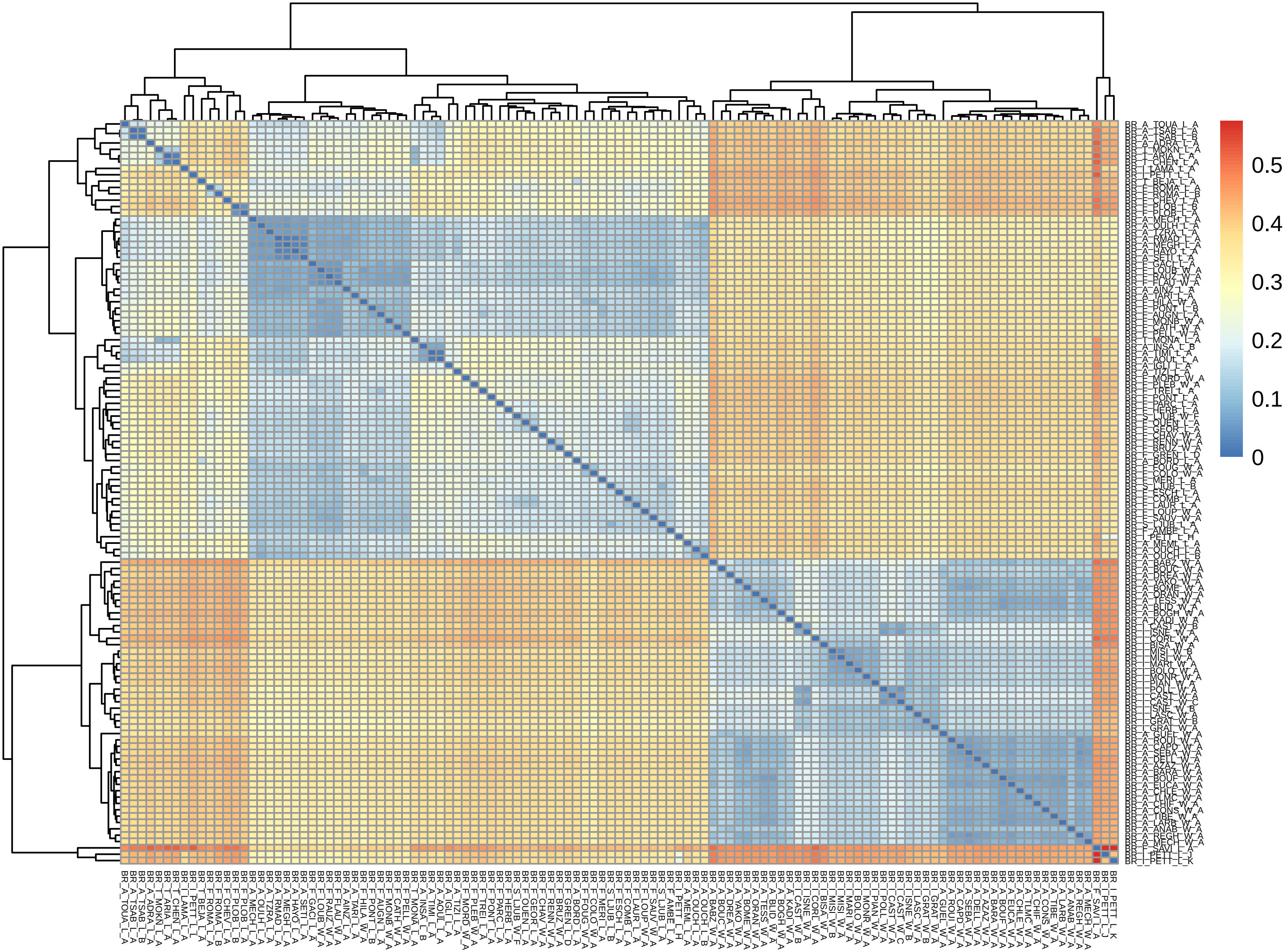
0.5
0.4
0.3
0.2
0.1
0

Supplementary Figure S2. Test showing that the Slovenian spontaneous population (WS) is closer to any landrace (Lx) than to the Algerian and Italian spontaneous populations (WA and WI), with the boxplot of the |Z-scores| for the three possible configurations including one WS, one WA, one WI and one landrace (Lx) (A) and the results of the tests of treeness (B). Test showing that the Slovenian spontaneous populations (WS) tend to be closer to Slovenian landraces (LS) or other landraces (Ly) than to other spontaneous populations (from Algeria or Italy, Wx), with the boxplot of the |Z-scores| for the three possible configurations including one WS, one LS, one spontaneous population and one landrace (C) and the results of the tests of treeness (D).

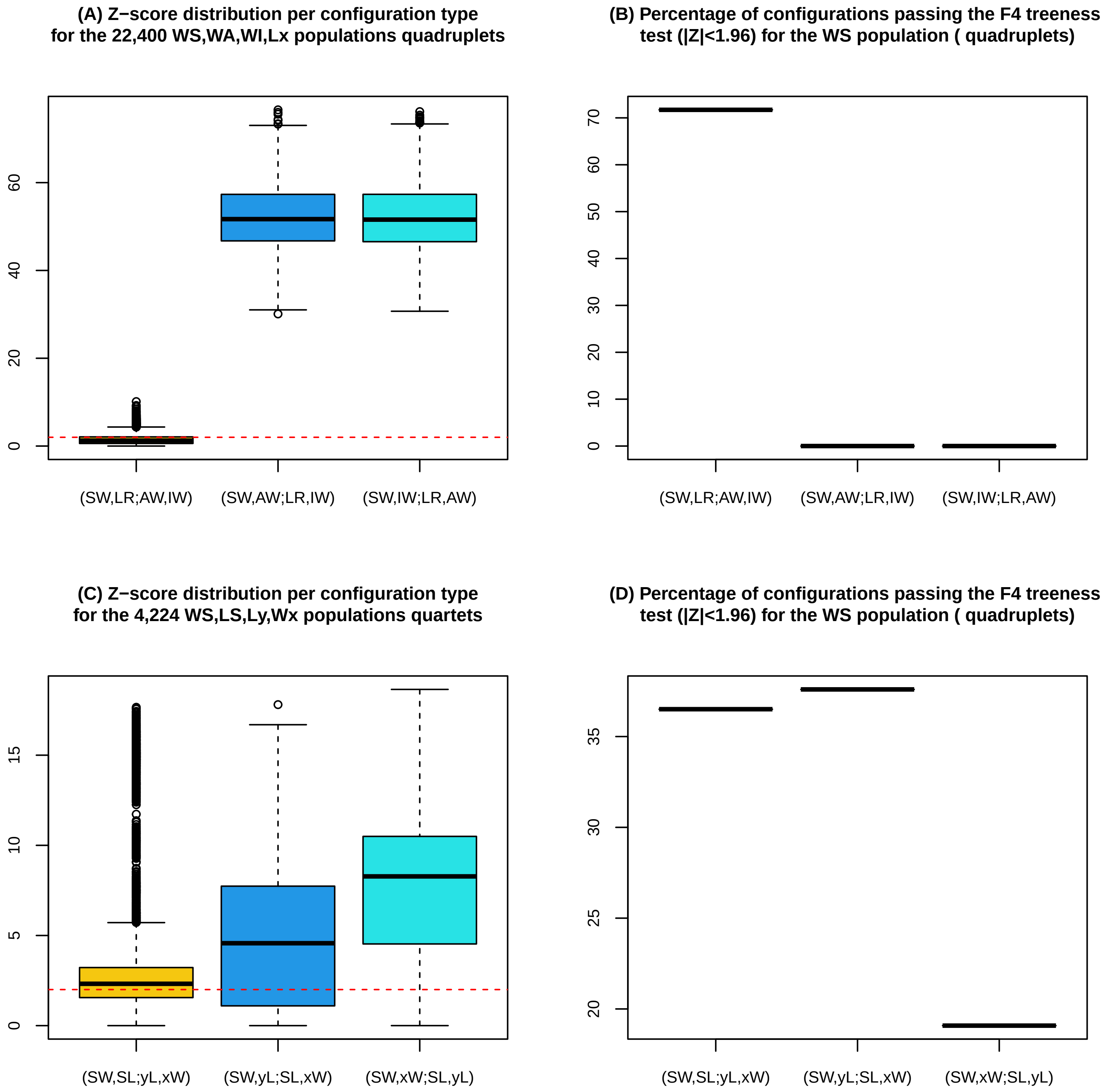
(A) Z−score distribution per configuration type
for the 22,400 WS,WA,WI,Lx populations quadruplets
0
20
40
60
(SW,LR;AW,IW)
(SW,AW;LR,IW)
(SW,IW;LR,AW)
(B) Percentage of configurations passing the F4 treeness
test (|Z|<1.96) for the WS population (quadruplets)
0
10
20
30
40
50
60
70
(SW,LR;AW,IW)
(SW,AW;LR,IW)
(SW,IW;LR,AW)
(C) Z−score distribution per configuration type
for the 4,224 WS,LS,Ly,Wx populations quartets
0
5
10
15
(SW,SL;yL,xW)
(SW,yL;SL,xW)
(SW,xW;SL,yL)
(D) Percentage of configurations passing the F4 treeness
test (|Z|<1.96) for the WS population (quadruplets)
20
25
30
35
(SW,SL;yL,xW)
(SW,yL;SL,xW)
(SW,xW;SL,yL)

Supplementary Figure S3. Plot of the Multiple Correspondence Analysis on qualitative morphological variables describing the root shape and colour (internal and external), showing individual coordinates on the dimensions 1 and 2 (A) and 3 and 2 (B). The codes for the quantitative variables used (root colour and shape) are detailed by the International Board for Plant Genetic Resources (IBPGR and CEC 1990).

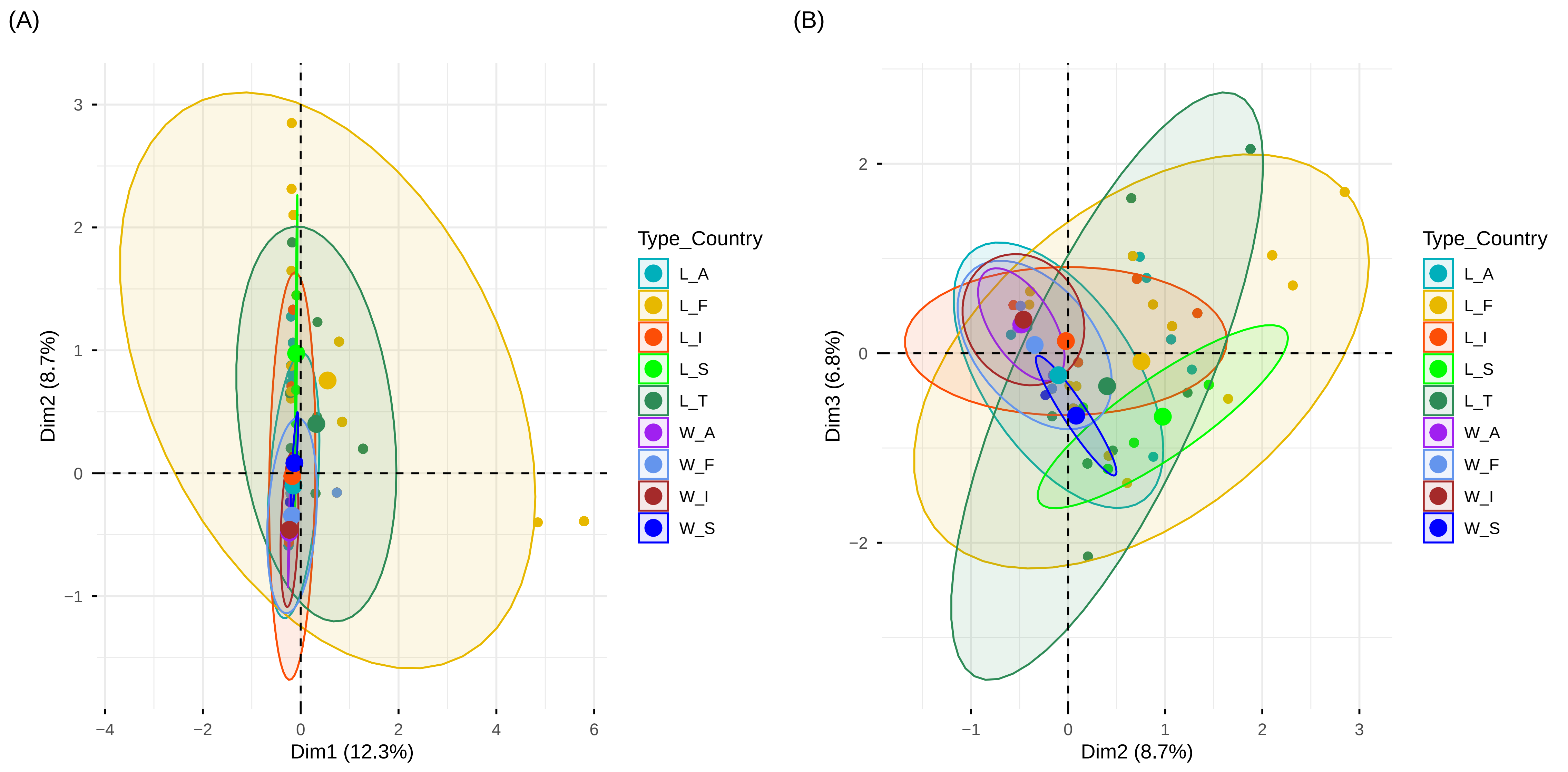
(A)
(B)
Dim1 (12.3%)
Dim2 (8.7%)
Dim2 (8.7%)
Dim3 (6.8%)
Type_Country
L_A
L_F
L_I
L_S
L_T
W_A
W_F
W_I
W_S
Type_Country
L_A
L_F
L_I
L_S
L_T
W_A
W_F
W_I
W_S

Supplementary Figure 4.

Comparison of root morphology in landraces (left column), feral populations (centre) and wild populations (right) from several countries. Each panel shows a photograph of the root (whole and cut) of one plant, which was collected from the common garden in Rennes. The names of the populations are written on the white labels.

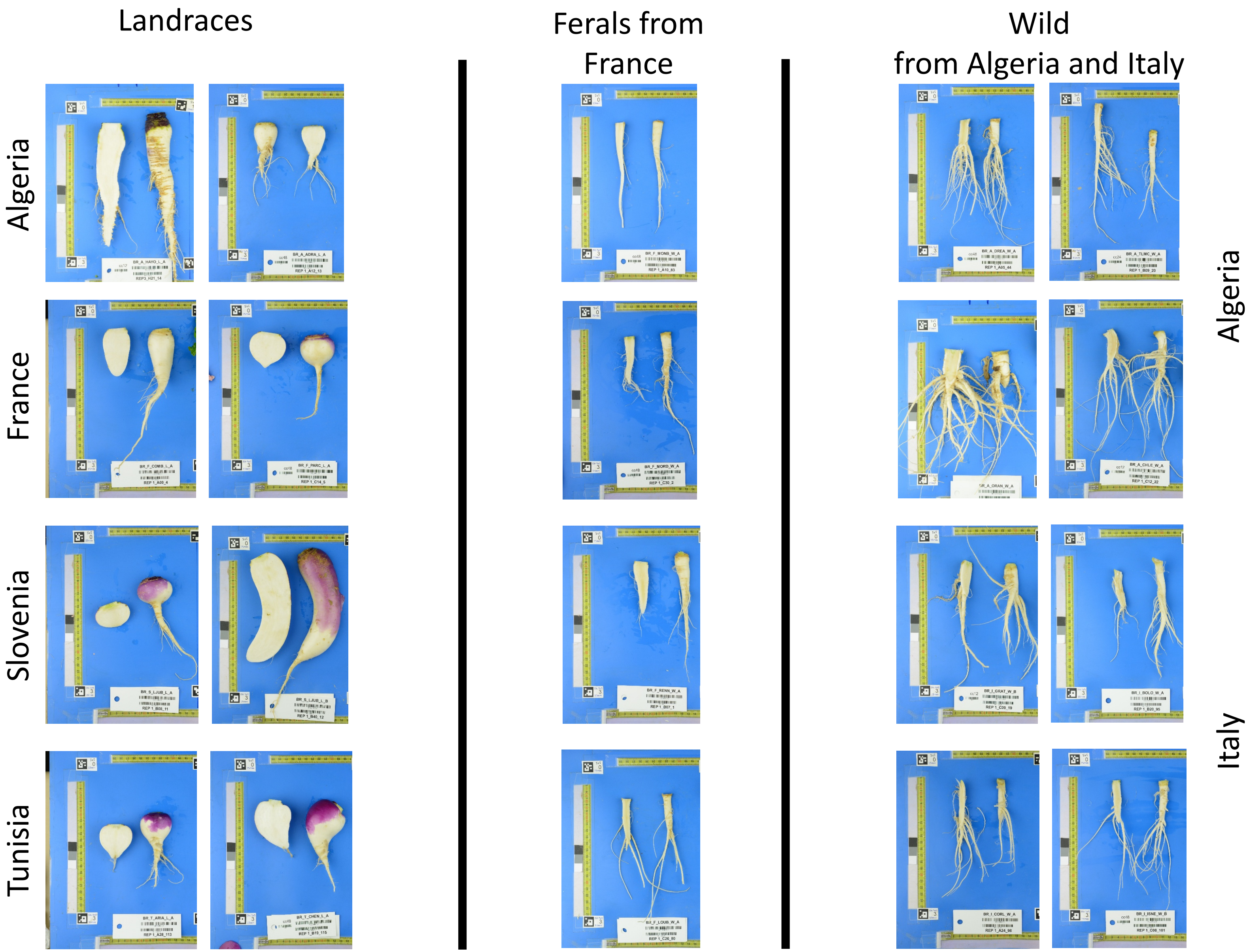

Supplement: Supplementary file 3 — Figure S1: Heatmap representing the genome‐wide estimates of FST coefficients for each of the population pairs, estimated using the compute.fstat() function in the R package poolfstat. The populations names are encoded as species_country_locality_type_replicate where species is BR for Brassica rapa ; country is A for Algeria, F for France, I for Italy, S for Slovenia and T for Tunisia; and type is L for Landraces and W for spontaneous. Figure S2: Test showing that the Slovenian spontaneous population (WS) is closer to any landrace (Lx) than to the Algerian and Italian spontaneous populations (WA and WI), with the boxplot of the |Z‐scores| for the three possible configurations including one WS, one WA, one WI and one landrace (Lx) (A) and the results of the tests of treeness (B). Test showing that the Slovenian spontaneous populations (WS) tend to be closer to Slovenian landraces (LS) or other landraces (Ly) than to other spontaneous populations (from Algeria or Italy, Wx), with the boxplot of the |Z‐scores| for the three possible configurations including one WS, one LS, one spontaneous population and one landrace (C) and the results of the tests of treeness (D). Figure S3: Plot of the Multiple Correspondence Analysis on qualitative morphological variables describing the root shape and colour (internal and external), showing individual coordinates on the dimensions 1 and 2 (A) and 3 and 2 (B). The codes for the quantitative variables used (root colour and shape) are detailed by the International Board for Plant Genetic Resources (IBPGR and CEC 1990). Figure S4: Comparison of root morphology in landraces (left column), feral populations (centre) and wild populations (right) from several countries. Each panel shows a photograph of the root (whole and cut) of one plant, which was collected from the common garden in Rennes. The names of the populations are written on the white labels. [file MEC-35-e70461-s004.pdf]
