# Supplementary material for: Genetic and Morphological Diversity in Spontaneous Populations of Brassica rapa: How Do Feral Populations Differ From Wild Ones?
Source: Mol Ecol. 2026 Jul 8;35(13):e70461. doi: 10.1111/mec.70461 (PMC13346339; doi:10.1111/mec.70461)
Supplement: Supplementary file 4 — Table S1: Type of land use for the spontaneous populations sampled in the wild in Algeria, France, Italy and Slovenia. The land use categories are arranged along a gradient of agricultural intensity ranging from cultivated fields or annual or perennial crops to semi‐natural or urban areas. [file MEC-35-e70461-s003.docx]

| **Type of land use** | **Cultivated** | **Algeria** | **France** | **Italy** | **Slovenia** | ***Total*** |
| --- | --- | --- | --- | --- | --- | --- |
| Annual crops | Yes | 2 |  | 1 | 1 | *4* |
| Perennial crops | Yes | 14 | 4 | 8 |  | *26* |
| Pasture | No |  | 1 | 1 |  | *2* |
| Wasteland or fallow | Yes | 1 |  |  |  | *8* |
|  | No | 2 | 1 | 2 |  |  |
|  | Not determined | 1 | 1 |  |  |  |
| Field border | No | 2 | 5 | 3 |  | *13* |
|  | Not determined | 2 | 1 |  |  |  |
| Semi-natural area (river/pond side, public garden) | Yes | 1 |  |  |  | *7* |
|  | No |  | 2 |  |  |  |
|  | Not determined | 3 | 1 |  |  |  |
| Others | No |  |  | 2 |  | *2* |
| *Total* |  | *28* | *16* | *17* | *1* | *62* |

**Supplementary Table 1.** Type of land use for the 62 spontaneous populations sampled in the wild in Algeria, France, Italy and Slovenia. The land use categories are arranged along a broad gradient of agricultural intensity ranging from cultivated fields of annual or perennial crops to semi-natural areas.
